# Amyloid Fibrils of the s36 Protein Modulate the Morphogenesis of *Drosophila melanogaster* Eggshell

**DOI:** 10.3390/ijms252312499

**Published:** 2024-11-21

**Authors:** Anna A. Valina, Vera A. Siniukova, Tatyana A. Belashova, Alexander A. Kanapin, Anastasia A. Samsonova, Alexey E. Masharsky, Anna N. Lykholay, Svetlana A. Galkina, Sergey P. Zadorsky, Alexey P. Galkin

**Affiliations:** 1 St. Petersburg Branch, Vavilov Institute of General Genetics, Russian Academy of Sciences, Universitetskaya Emb. 7/9, 199034 St. Petersburg, Russia; annavalina@mail.ru (A.A.V.); veleenna@yandex.ru (V.A.S.); trbmc@mail.ru (T.A.B.); zadorsky@mail.ru (S.P.Z.); 2 Department of Genetics and Biotechnology, Faculty of Biology, St. Petersburg State University, Universitetskaya Emb. 7/9, 199034 St. Petersburg, Russia; svetlana.galkina@mail.ru; 3 Laboratory of Amyloid Biology, St. Petersburg State University, Universitetskaya Emb. 7/9, 199034 St. Petersburg, Russia; 4 Center for Computational Biology, Peter the Great St. Petersburg Polytechnic University, Polytehnicheskaya Str. 29, 195251 St. Petersburg, Russia; a.kanapin@gmail.com (A.A.K.); a.samsonova@spbu.ru (A.A.S.); 5 The “Bio-Bank” Resource Center, Research Park of St. Petersburg State University, Universitetskaya Emb. 7/9, 199034 St. Petersburg, Russia; masharsky@gmail.com; 6 Research Resource Center “Molecular and Cell Technologies”, Research Park of St. Petersburg State University, Botanicheskaya Str. 17, Petergof, 198504 St. Petersburg, Russia; lankira@mail.ru

**Keywords:** *Drosophila*, functional amyloid, s36, morphogenesis, eggshell, chorion, sterility, gene *CG33223*

## Abstract

*Drosophila melanogaster* is the oldest classic model object in developmental genetics. It may seem that various structures of the fruit fly at all developmental stages have been well studied and described. However, recently we have shown that some specialized structures of the *D. melanogaster* eggshell contain an amyloid fibril network. Here, we demonstrate that this amyloid network is formed by the chorionic protein s36. The s36 protein colocalizes with the amyloid-specific dyes Congo Red and Thioflavin S in the micropyle, dorsal appendages, and pillars. The fibrils of s36 obtained from the eggs demonstrate amyloid properties. In the context of the *CG33223* gene deletion, the s36 protein is produced but is not detected in the eggshell. The absence of amyloid fibrils of s36 in the eggshell disrupts the endochorion morphology and blocks the development of the micropyle, dorsal appendages, and pillars, leading to sterility. Our data show for the first time that amyloid fibrils are essential for morphogenesis modulation. We suggest that attachment of follicle cells to the s36 extracellular fibrils triggers signaling to enable subsequent cellular divisions needed for building the specialized eggshell structures.

## 1. Introduction

The *Drosophila melanogaster* eggshell is produced at the late stages of oogenesis by epithelial follicle cells (FCs) and is assembled as a highly organized multi-layered structure exhibiting regional and radial complexity [1]. This structure allows sperm penetration, protecting the growing embryo from external aggressions and at the same time ensuring gas exchange [2].

**Figure 1:**
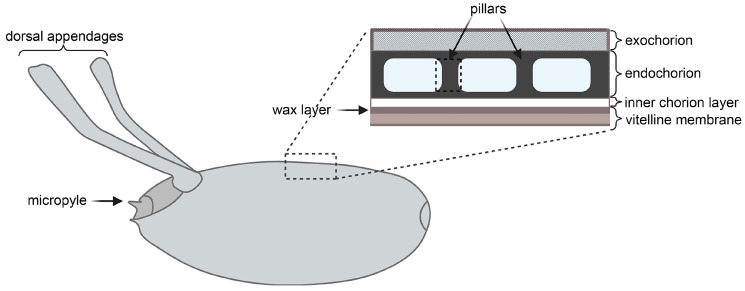
Structural organization of the *Drosophila melanogaster* eggshell (the figure is based on Figure 1 from [2] with modifications).

The eggshell consists of two main layers (Figure 1)—the internal vitelline membrane layer and the external chorion layer [3,4,5]. The chorion is highly structured and consists of a wax layer, inner chorion layer, endochorion, and exochorion [2]. It is formed at stages 10–14 of egg development from FC secretion. FCs differentiate into several specialized groups in response to positional signals under control of the bone morphogenetic protein (BMP), epidermal growth factor (EGF), Jun-N-terminal kinase (JNK), and Notch pathways [6,7,8]. Apart from the organization of chorion morphology, FC migration and division also determine the formation of specialized structures such as dorsal appendages and the micropyle. Dorsal appendages ensure egg buoyancy in an aquatic environment and are also needed for gas exchange [7]. The micropyle is a cone-shaped protrusion at the anterior pole through which spermatozoa penetrate to fertilize the egg [2]. These structures are built by distinct populations of FCs that execute cell-specific secretory programs. The structures called pillars form in the endochorion region and delimit air spaces that allow the egg to facilitate gas exchange. At the final stage of egg development, the FCs die, and the endochorionic-hardening process occurs.

In accordance with the development program, distinct FC populations secrete polysaccharides and certain proteins that make up the chorion. In the fruit fly, six major and several minor chorion proteins have been identified. All the genes coding for these proteins are located at two chorion loci on chromosomes X and 3 [9,10]. Two clusters of chorion genes are amplified in FCs, which ensures a high level of production of chorion-forming proteins [11]. Alterations in the expression of the genes encoding major proteins of the chorion cause morphological defects in its architecture. For example, downregulation of the *Cp36* and *Cp38* genes encoding the s36 and s38 proteins, respectively, leads to morphological abnormalities of the endochorion, blocks the endochorionic-hardening process, and promotes the formation of reduced dorsal appendages [12,13]. Moreover, downregulation of the *Cp36* gene leads to impairment of the micropyle, resulting in egg sterility [12].

It seems that the structural components and morphology of the eggshell have been described in detail. In this regard, the recent discovery of an amyloid fibril network in some structures of the eggshell in such a well-studied object as *D. melanogaster* [14] was a complete surprise. We found that the micropyle, dorsal appendages, and pillars of *D. melanogaster* eggs can be stained with the amyloid-specific dyes Congo Red (CR) and Thioflavin S (ThS) [14]. The amyloid fibrils are often associated with various incurable pathologies, but some proteins of higher eukaryotes normally function in the amyloid form [15,16]. They have been identified in bacteria, yeast, plants, and animals and perform structural, storage, and protective functions. For example, it was recently shown that amyloid fibrils of the Orb2 protein bind RNA molecules in the neurons of the fruit fly brain [17]. Here, we identify the protein that forms the amyloid fibrils in the fruit fly eggshell, determine the gene responsible for its localization and aggregation, and discuss the biological role of these amyloid structures.

## 2. Results

### 2.1. Proteomic Screening for Amyloid-like Proteins in D. melanogaster Eggs

We had previously shown that specialized structures of the *D. melanogaster* eggshell can be stained with the amyloid-specific dyes CR and ThS [14]. In order to identify a protein with amyloid properties in fruit fly eggs, we used the universal method of proteomic screening for amyloids [18,19]. All known amyloids form fibrils that are resistant to Sodium Dodecyl Sulfate (SDS) treatment at room temperature (RT). The method of proteomic screening makes it possible to separate high-molecular-weight SDS-resistant protein aggregates from other proteins and identify them using mass spectrometry. The protein lysate from eggs of the Oregon-R strain was treated with 1% SDS, and the fraction of high-molecular-weight aggregates was separated from other proteins. In the next step, the protein lysate was trypsinized, the peptides were separated using high-resolution chromatography, and they were identified by mass spectrometry (Table 1 and Figures S1–S6). The s36 protein and five other chorionic proteins were identified with the highest score of mass spectrometry.

The ability to form SDS-resistant aggregates and complexes is characteristic not only of amyloids [20]. Moreover, chorionic proteins can be detected in the fraction of SDS-resistant aggregates due to the fact that chitin-like polysaccharides act like glue to bind all chorionic proteins into a single structure in the eggshell. Chorionic mucopolysaccharides are known to contain the amino sugar glucosamine [21], which is a target for chitinase. Therefore, we treated the protein lysate from fruit fly eggs with chitinase, which destroys the polysaccharide backbone, and repeated proteomic screening for the proteins that form SDS-resistant aggregates. After chitinase treatment, the s36 protein was still detected with the highest mass spectrometry score. In addition, three proteins (s19, s38, and s15) were detected in the SDS-resistant fraction, although with significantly lower scores than before treatment with chitinase (Table 1 and Figures S7–S10).

Thus, based on the results of proteomic screening, it can be assumed that the s36 protein is the most promising candidate for the functional amyloid role in fruit fly eggs.

### 2.2. Chromosomal Rearrangement at the 7F Locus of the X Chromosome Prevents the Formation of Amyloid Structures in the Fruit Fly Eggshell

**Figure 2:**
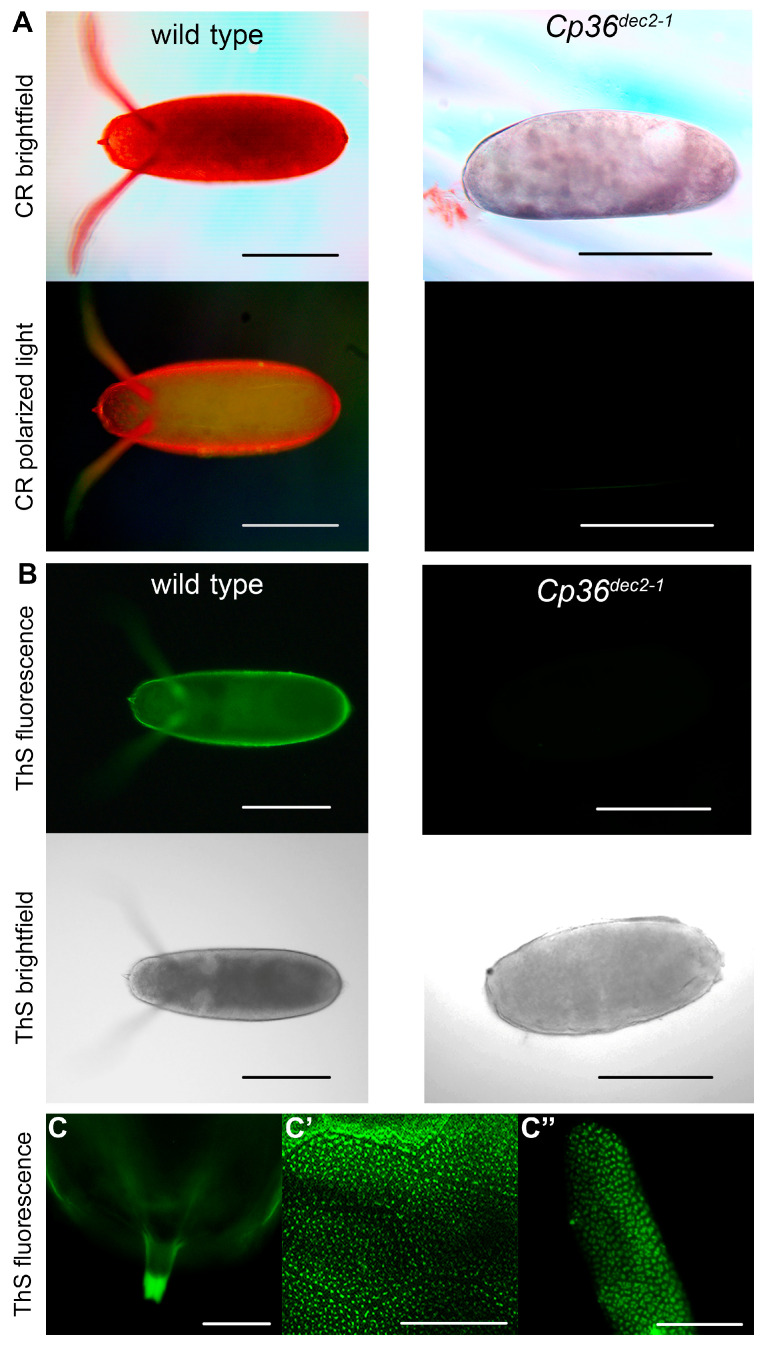
CR and ThS staining of the eggshell of the wild-type flies and the flies homozygous for the *Cp36^dec2−1^* chromosomal rearrangement. (**A**) Staining of the fruit fly eggs with CR in brightfield and polarized light. (**B**) Staining of fruit fly eggs with ThS under UV light and in brightfield. (**C**–**C**″) Staining of the micropyle (**C**), the pillars (**C′**), and the modified pillars of the dorsal appendages (**C**″) with ThS (green fluorescence under UV light). Scale bars, 200 µm (**A**,**B**); 20 µm (**C**–**C**″).

According to our cytological data, amyloid-specific dyes stain the eggshell structures, such as the micropyle, dorsal appendages, and pillars [14]. These eggshell structures are known to not form in the context of the chromosomal rearrangement *Cp36^dec2−1^* in the 7F locus of the X chromosome [22]. The *Cp36^dec2−1^* rearrangement has not been characterized so far but is assumed to be a strong hypomorphic mutation of the *Cp36* gene encoding the s36 protein. This chromosomal rearrangement in a homozygote has been proven to be lethal and causes disruption in the eggshell structure. We compared CR and ThS staining patterns of the eggshells of wildtype and mutant flies homozygous for this chromosomal rearrangement. The eggshells of the wild-type females stain with amyloid-specific dyes and exhibit yellow-green birefringence when stained with CR (Figure 2A–C). This is a common feature of all known amyloids. At a high magnification, it is clearly visible that the amyloid-specific dye stains only specialized structures rather than the entire surface of the eggshell (Figure 2C–C″). Only the tip of the micropyle, the modified pillars of the dorsal appendages, and the pillars in the main body of the chorion are positive for the amyloid-specific dye. The eggshells of females homozygous for the *Cp36^dec2−1^* chromosomal rearrangement did not bind amyloid-specific dyes, in contrast to the eggshells of the wild-type flies (Figure 2A,B). The eggshells of the females homozygous for the chromosomal rearrangement were more rounded and had neither a formed micropyle nor dorsal appendages (Figure 2A,B). A small percentage (about 5%) of eggs with vestigial dorsal appendages or unformed projections in the micropyle region were found (Figure S11). Moreover, the mutant chorion was not as dense as that of the wild type. These results indicate that the *Cp36^dec2−1^* chromosomal rearrangement disrupts the production or secretion of s36 or some other amyloidogenic proteins, the genes of which are located in the 7F locus of the X chromosome.

### 2.3. The s36 Protein Forms Insoluble Aggregates and Colocalizes with the Amyloid-Specific Dye ThS in the Fruit Fly Eggshell

Our data suggest that amyloid structures in the fruit fly eggshell can be formed by the s36 protein. To test this hypothesis, we extracted RNA from the eggs of females of the Oregon-R strain, synthesized the *Cp36* cDNA, produced the full-length protein in *Escherichia coli*, and obtained the polyclonal primary antibodies against the s36 protein. These antibodies were used for immunochemical and immunocytological detection of the s36 protein.

**Figure 3:**
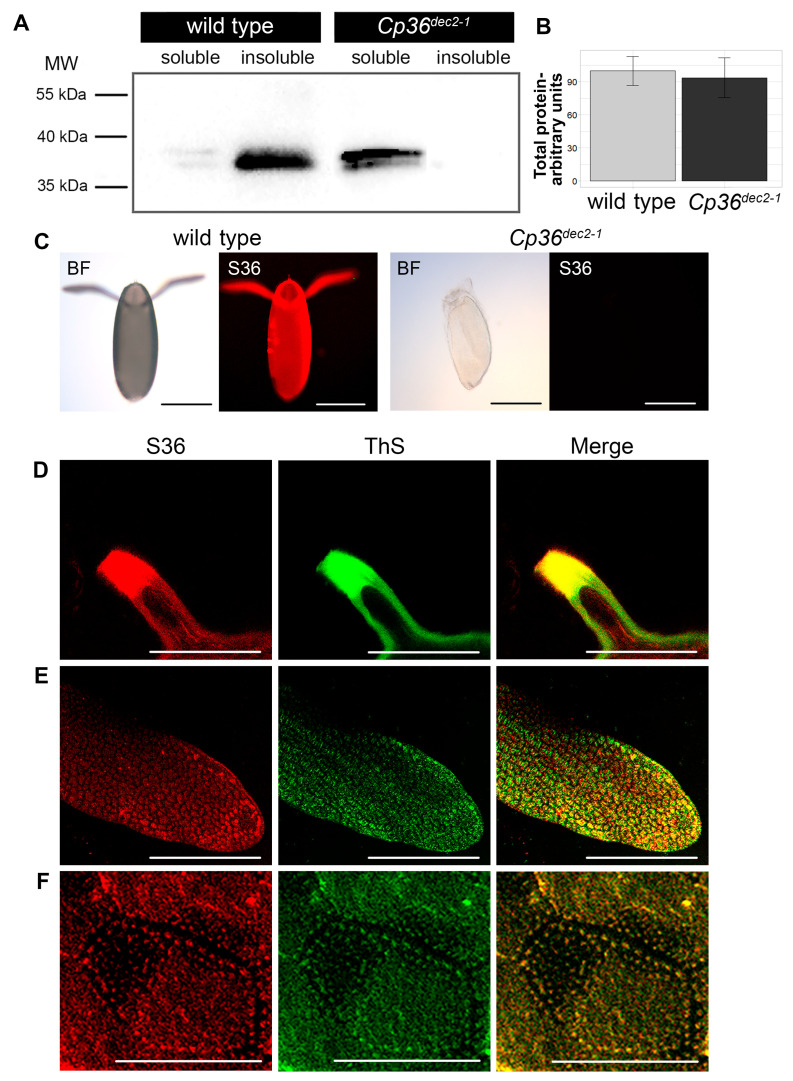
Immunodetection of the s36 protein in the fruit fly egg lysates and intact eggshells. (**A**) Immunoblotting of the s36 protein obtained from the egg lysates of the wild-type and mutant flies homozygous for the *Cp36^dec2−1^* chromosomal rearrangement. The protein lysates were separated into soluble and insoluble fractions by low-speed centrifugation. The s36 protein was detected in the insoluble fraction in the Oregon-R eggs and in the soluble fraction of the mutant eggs homozygous for the *Cp36^dec2−1^* chromosomal rearrangement. (**B**) Densitometric quantification of the data is shown in A. The relative intensities of the bands corresponding to S36 are presented as the mean ± SEM of three independent egg lysate samples. (**C**) Staining of the Oregon-R and the mutant eggs with the s36-specific antibodies. The s36-specific antibodies bind the eggshell of the Oregon-R flies (red fluorescence) but do not bind the eggshell of the mutant flies homozygous for the *Cp36^dec2−1^* chromosomal rearrangement. (**D**–**F**) Staining of the micropyle, the pillars, and the dorsal appendages of the Oregon-R eggs with the s36-specific antibodies (red) and ThS (green). The s36-specific antibodies bind the micropyle (**D**), the pillars (**E**), and the modified pillars in the dorsal appendages (**F**) and colocalize with the amyloid-specific dye ThS. Scale bars, 200 µm (**C**); 20 µm (**D**–**F**).

The protein lysates obtained from the ovaries of females of the Oregon-R strain and from the females homozygous for the *Cp36^dec2−1^* chromosomal rearrangement were separated into soluble and insoluble fractions by low-speed centrifugation. Using Western blotting, we showed that s36 is produced both in the eggs of the wild-type strain and in the mutant strain eggs (Figure 3A). The average value of the s36 signal intensity (in arbitrary units) was 100.00 ± 13.1 and 93.75 ± 18.1 in wild-type and mutant eggs, respectively (*p*-value > 0.05 (0.8)). Thus, no differences in the level of the s36 protein production were detected (Figure 3B). However, the s36 protein formed insoluble aggregates in the eggs of the Oregon-R females, while in the mutant eggs it was present in the soluble fraction. As an internal control, we showed that another major chorionic protein, s38, is also produced in eggs from both wild-type and mutant females. However, the level of aggregation of this protein in the eggs of females of these lines does not differ (Figure S12A). Moreover, the s36-specific antibodies did bind the eggshell of the wild-type flies, yet they were not detected in the eggshell of the mutant eggs (Figure 3C). The control protein s38 was detected in the egg membrane of both wild-type and mutant females (Figure S12B). It is known that s36 is produced in FCs and then secreted into the chorion [23]. Our data show that s36 is produced in the eggs of both wild-type and mutant flies, but in the context of the *Cp36^dec2−1^* chromosomal rearrangement, this protein does not aggregate and is not detected in the eggshell.

We analyzed the colocalization of the s36-specific antibody and amyloid-specific dye ThS in the eggshell of the Oregon-R females (Figure 3D–F). The s36-specific antibodies colocalized with the amyloid-specific dye and stained the tip of the micropyle, the modified pillars in the dorsal appendages, and the pillars in the main body of the eggshell. Thus, we can conclude that s36 aggregates form amyloid fibrils in the specialized structures of the fruit fly eggshell.

### 2.4. The CG33223 Gene Affects the Localization and Aggregation of the s36 Protein into the Eggshell

Impaired secretion of the s36 protein in the flies carrying the *Cp36^dec2−1^* chromosomal rearrangement may be associated with a mutational change affecting the predicted N-terminal signal sequence of s36 (20 aa). We compared the cDNA sequences of the *Cp36* transcripts from the Oregon-R and mutant females and found them to be identical (Figure S13) and not differ from the reference sequence presented in the FlyBase database (FBgn0000359; https://flybase.org/, accessed on 19 April 2024). Therefore, our results show that the chromosomal rearrangement blocking the relocation of the s36 protein into the eggshell does not affect the gene encoding this protein.

**Figure 4:**
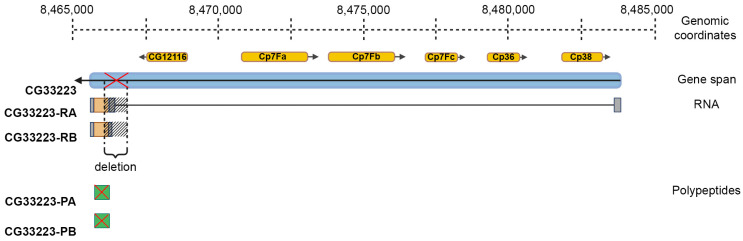
Structure of the *CG33223* gene and its products in the *Drosophila melanogaster* strain BDSC #4842 (chromosome X) with the *Cp36^dec2−1^* chromosomal rearrangement. The direction of transcription is indicated by a black arrow. The open reading frame is indicated in brown color. The exons are indicated as gray rectangles, and the intron is a black line. The boundaries of the deletion are indicated by a hatching and by a red cross. The polypeptide produced in wild-type flies is indicated in a greenish color. Also, a red cross indicates that the polypeptide is not formed against the background of deletion. The IDs of the *CG33223* products were obtained from the FlyBase database (https://flybase.org/, accessed on 10 May 2024).

To analyze the *Cp36^dec2−1^* chromosomal rearrangement, we performed comparative sequencing of genomic DNA from the wild-type flies of the Oregon-R strain and flies homozygous for the *Cp36^dec2−1^* chromosomal rearrangement (BioProject ID: PRJNA1031583). The 12.6 × 10^6^ pair-end reads (3.8 Gb) for the DNA sample from the Oregon-R strain and the 17.8 × 10^6^ pair-end reads (5.4 Gb) for the DNA sample from the mutant flies were obtained. We performed a search for structural variants using the Delly and GridSS algorithms as described in Materials and Methods. Both tools show that the *Cp36^dec2−1^* chromosomal rearrangement is a deletion in the 7F locus of the X chromosome that affects the *CG33223* gene with unknown function (Figure S14). Using Sanger sequencing, we showed that the coordinates for the genomic location of this deletion are chrX: 8,466,141–8,466,777 (deletion of 637 bp; GenBank ID: PP658205). The *CG33223* gene has two transcripts with a common reading frame encoding identical polypeptides. The deletion begins before the first coding triplet and completely blocks the synthesis of this polypeptide (Figure 4 and Figure S14). All other differences between the genomes of the mutant and the wild-type flies in the 7F locus reflect the natural polymorphism and do not lead to disruption of other reading frames. Based on the results obtained, we can conclude that the chromosomal rearrangement *Cp36^dec2−1^* disrupts the *CG33223* gene expression. These data suggest that the product of this gene directly or indirectly modulates the secretion of the s36 protein within the eggshell.

### 2.5. The s36 Protein Forms Amyloid Fibrils In Vitro and in the Fruit Fly Eggshell

**Figure 5:**
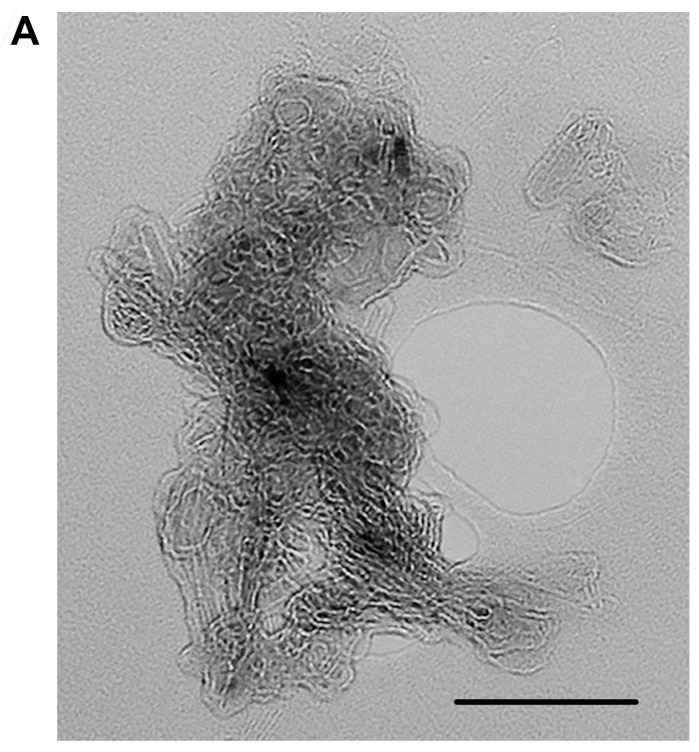

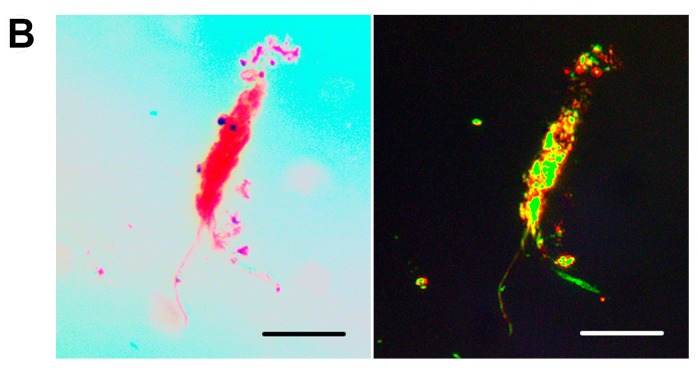
Fibrils immunoprecipitated with antibodies against the s36 protein from the Oregon-R eggs. (**A**) An electron micrograph of fibrils stained with uranyl acetate. (**B**) CR staining of the s36 protein immunoprecipitated from the Oregon-R eggs. The left panel is brightfield (red), and the right panel is polarized light (apple-green). Scale bars, 100 nm (**A**); 20 µm (**B**).

To confirm the amyloid properties of the s36 protein, we extracted the fibrils of this protein from the ovaries of the Oregon-R strain by immunoprecipitation and analyzed them using electron and light microscopy. For this purpose, the previously described method for immunoprecipitation of amyloid fibrils from various organisms was used [19,24]. The fibrils of s36 were visualized with transmission electron microscopy (TEM) using negative contrast staining (Figure 5A). The extracted fibrils bound CR and exhibited yellow-green birefringence after this staining (Figure 5B), which is typical for all known amyloids.

We also analyzed the amyloid properties of s36 in vitro. The 6His-tagged s36 protein was produced in *E. coli* and purified and incubated for 5 days in phosphate-buffered saline (PBS). Fibril formation was verified by TEM and CR staining (Figure S15).

Thus, in addition to staining with amyloid-specific dyes in vivo, we demonstrated that s36 forms amyloid fibrils in the fruit fly eggshell and in vitro.

## 3. Discussion

In this study, we present findings that in *D. melanogaster*, the s36 protein can form amyloid fibrils in vivo and in vitro; the absence of s36 in the eggshell prevents its amyloid aggregation, disrupts endochorion morphology, and inhibits the development of the micropyle, dorsal appendages, and pillars. This is the first report that an amyloid fibrillar structure is essential for morphogenesis. Our data suggest that the s36 protein forms extracellular amyloid fibrils after secretion from FCs into the eggshell of *D. melanogaster*. The fibrils of s36 extracted from eggs as well as the fibrils obtained in vitro were detected by electron microscopy and exhibited yellow-green birefringence in polarized light after CR staining.

Immunostaining with the s36-specific antibodies revealed the presence of this protein in the tip of the micropyle, the pillars in the endochorion, and the modified pillars in the dorsal appendages—foci that stain with amyloid-specific dyes (Figure 3D–F). It is known that the micropyle tip is formed by the border FCs [2,25], while the rest of the micropyle and the sperm channel are formed by the polar non-migratory FCs [26,27]. Obviously, s36 is produced only in the border cells and not in the polar non-migratory cells.

The chromosome rearrangement *Cp36^dec2−1^* affects the localization and aggregation of the s36 protein. We show that this chromosomal rearrangement *Cp36^dec2−1^* is the deletion disrupting the expression of the *CG33223* gene in the X chromosome (Figure 4). The functions of the *CG33223* gene remain obscure. It is only known that the product of this gene has a ubiquitin-like domain. Orthologs of this protein have been found only in some species of *Drosophilidae*, and their functions are also unknown. Currently we cannot ascertain whether *CG33223* regulates secretion of the s36 protein directly or indirectly.

The s36 protein is produced in follicular cells and then secreted from them into the outer shell of the oocyte during the final stages of oogenesis [4]. Earlier, it was shown that the RNAi-mediated 50-fold decrease in the level of s36 production caused some disturbances in the morphology of the chorion [12]. In particular, downregulation of s36 production leads to the development of reduced appendages with defective morphology and causes the formation of a micropyle missing the channel for sperm entry. Our data show that in the context of the chromosomal rearrangement *Cp36^dec2−1^*, the s36 protein is produced in the egg (Figure 3A) but not detected in the eggshell (Figure 3C). This event is even more critical for the eggshell morphology than the RNAi-mediated downregulation of *Cp36* expression. Dorsal appendages in the *Cp36^dec2−1^* mutants are either absent or are rudimental and collapse up during egg laying. Under such chromosomal rearrangement, the micropyle does not form at all (Figure 2A,B). The formation of these structures in wild-type eggs is determined by the migration and division of specialized FCs under control of the BMP, EGF, JNK, and Notch pathways [7,8]. The cells that give rise to the dorsal appendages begin to form blind tubular structures with an internal cavity at stage 11 of egg development [8]. The appendages are formed after the secretion of chorionic proteins into the tube lumens. The absence of s36 in the eggshell, most likely, prevents the division of the cells forming the dorsal appendage and the micropyle. Thus, the fibrils of the s36 protein are a component of the extracellular matrix, which is essential for the division of the cells forming specialized eggshell structures, such as the dorsal appendages and the micropyle. In this way, the s36 extracellular amyloid fibrils are an important element of the developmental program of the fruit fly eggshell. We assume that specialized FCs attach to the s36 amyloid fibrils, and this interaction triggers signaling to enable subsequent cellular divisions. In mammals, binding of the extracellular protein fibronectin to fibroblasts is required for their subsequent division in a similar manner [28].

Our data suggest that the s36 protein forms amyloid fibrils only in the eggshell. It is likely that the polysaccharides or hydrogen peroxide in the chorion contributes to the amyloid aggregation of this protein. The endochorionic-hardening process is triggered by hydrogen peroxide activating peroxidase, which promotes protein crosslinking mediated by the formation of di- and tri-tyrosine bonds [29]. At the same time, our results and the previously published data [12] indicate that the absence of s36 in the chorion disrupts its hardening. These data suggest that the formation of s36 amyloid fibrils is one of the elements of the cascade triggering the hardening process.

**Figure 6:**
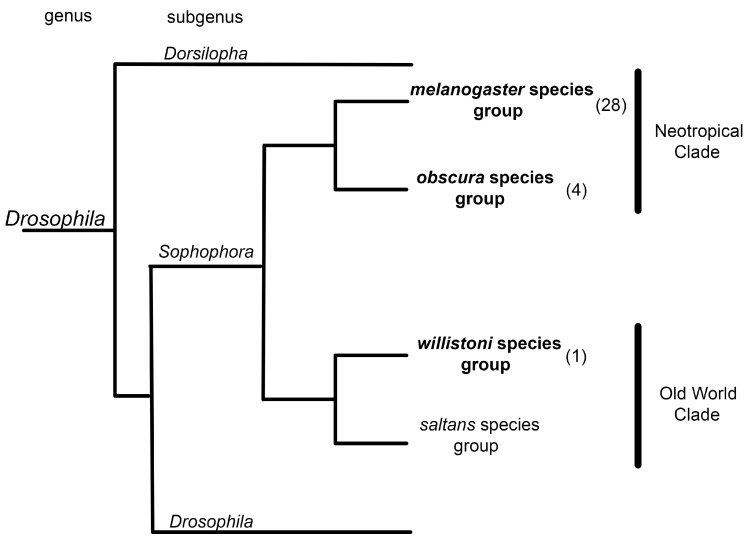
Distribution of potentially amyloidogenic region of the s36 protein across the phylogenetic tree of the genus *Drosophila*. Group representatives that have the s36 amyloidogenic sequence are in bold (only within the subgenus *Sophophora*). The number of representatives is indicated in brackets.

Amyloid fibrils of the s36 protein have been found in the pillars of the endochorion, the modified pillars of the dorsal appendages, and the tip of the micropyle. Why did amyloid structures arise in the eggshell and persist during evolution? We believe that these fibrils are an ideal material delimiting the cavities necessary for gas exchange, as well as the cavity of the channel for sperm entry. The s36 protein is specific to the *Schizophora* section of the *Diptera* order and is not found among other animals. Using the ArchCandy algorithm [30], we identified a potentially amyloidogenic region of this protein containing the sequence from the 48th to the 64th amino acid (Figure S16). The s36 amyloidogenic sequence is conserved and present in many representatives of the subgenus *Sophophora* of the genus *Drosophila* (33 species including *D. melanogaster*) (Figure 6), according to the BLAST algorithm (https://blast.ncbi.nlm.nih.gov/Blast.cgi, accessed on 2 July 2024). Apparently, the amyloid properties of the s36 protein emerged at the dawn of the appearance of the *Sophophora* subgenus around 35–50 million years ago and were picked up by natural selection. Proteins that form functional amyloid fibrils have also been found in the silkworm chorion and in the shell of mammalian oocytes [31,32,33]. The proteins forming such amyloid fibrils in the shell of insect eggs and mammalian oocytes have no similarity. They arose repeatedly and independently in the course of evolution.

To summarize, we have shown that the chorion protein s36 forms an amyloid network in the fruit fly eggshell. The extracellular amyloid fibrils of s36 are essential for the endochorionic-hardening process, as well as for the formation of the pillars, the micropyle, and the dorsal appendages. We suggest that the *CG33223* gene regulates, either directly or indirectly, the secretion of the s36 protein from FCs into the eggshell. Therefore, extracellular amyloid fibrils of s36 are a morphogenetic factor modulating the development of the fruit fly eggshell.

## 4. Materials and Methods

### 4.1. Fly Strains

*D. melanogaster* flies were maintained using standard methods on a standard fly medium at 25 °C. The Oregon-R strain was used as a wild-type control. The strain BDSC, #4842 (BL:4842), with an uncharacterized chromosomal rearrangement in the 7F region of the X chromosome, *y[1] cv[1] Cp36[dec2-1] v[1] f[1]/FM0*, was obtained from the Bloomington Drosophila Stock Center (https://bdsc.indiana.edu/, accessed on 15 November 2023). Females of the Oregon-R strain and of the BDSC, #4842 strain, homozygous for the *Cp36^dec2−1^* chromosomal rearrangement, aged 5–6 days, were used for experiments. The selection of flies was carried out using ether anesthesia. Eggs ready to be laid and whole ovaries were collected to study the chorion.

### 4.2. Proteomic Screening and Identification of Proteins Forming Amyloid-like Aggregates

Proteomic screening and the identification of proteins forming amyloid-like aggregates in oocytes of *D. melanogaster* were performed using the PSIA–LC–MALDI approach [18,19]. Fly ovaries were homogenized using a cryogenic laboratory mill, the Large Freezer/Mill 6870 (SPEX SamplePrep, Metuchen, NJ, USA), at −196 °C in liquid nitrogen and then stored at −70 °C. The homogenized ovarian tissue was suspended in Tris-buffered saline (TBS) (75 mM Tris-HCl at pH 7.6, 125 mM NaCl, 2 mM PMSF, 10 mM EDTA, 1 × Complete Protease Inhibitor Cocktail [Roche, Basel, Switzerland]). The lysate was clarified by centrifugation at 805× *g* for 5 min at 4 °C then resuspended in TBS and incubated for 30 min at RT with 5 mg RNase A (75–150 U/mg) (Thermo Fisher Scientific, Waltham, MA, USA). Then, lysate was ultracentrifuged at 223,321× *g* for 2 h at 8 °C through a 25% sucrose–TBS cushion. The aggregate-containing pellet was resuspended in TBS and treated with 1% SDS for 8 h at 18 °C. Then, the fraction of detergent-resistant protein complexes was separated by ultracentrifugation at 223,321× *g* for 8 h at 18 °C using a 20% sucrose–TBS cushion with 0.1% SDS. The resulting pellets were suspended in water and sedimented again at 223,321× *g* for 2 h at 8 °C. The obtained pellet was denatured in SDS-PAGE loading buffer (4 × buffer: 100 mM Tris-HCl at pH 6.8, 20% 2-mercaptoethanol, 8% SDS, 0.2% bromophenol blue, 40% glycerol) for 15 min at 95 °C. Then, detergents and salts were removed from the samples using HiPPR^TM^ Detergent Removal Spin Columns (Thermo Fisher Scientific, Waltham, MA, USA) and using PD SpinTrap^TM^ G-25 Desalting Columns (Cytiva, Marlborough, MA, USA) according to the manufacturer’s protocol. After overnight trypsinolysis at 37 °C, the peptide mixtures (1 μL) were loaded onto the Acclaim PepMap 300 HPLC reverse-phase column (150 mm, 75 μm, particle size 5 μm; Thermo Fisher Scientific, Waltham, MA, USA). They were separated in an acetonitrile gradient (2–90%) for 45 min using the UltiMate 3000 UHPLC RSLC nano-high-performance nanoflow liquid chromatograph (Dionex, Sunnyvale, CA, USA). Peptide fractions were collected every 10 s and loaded onto a 384-sample MTP AnchorChip 800/384 microtiter plate (Bruker Daltonics, Billerica, MA, USA) using the Proteineer fc II spotter (Bruker Daltonics, Billerica, MA, USA). Peptides were identified using an Ultraflextreme MALDI-TOF/TOF mass spectrometer (Bruker Daltonics, Billerica, MA, USA). MS spectra for each peptide fraction were determined and analyzed using WARP-LC software version 1.3. All raw files were processed with Mascot software version 2.4.2 using standard settings and searched against the UniProt database (https://www.uniprot.org/, accessed on 19 December 2023). α-Cyano-4-hydroxycinnamic acid was used as a matrix. The search was conducted with a mass tolerance of 100 ppm for the precursor ion and 0.9 Da for the fragment. Carboxymethylation of cysteine, partial oxidation of methionine, and one omitted trypsinolysis site were considered as valid modifications. Proteomic screening with chitinase treatment was performed similarly but with the addition of chitinase (20 µg) along with RNase A.

### 4.3. Preparation of Eggs for Histological Staining

The eggs of *D. melanogaster* at stage 14 were separated and placed on a glass slide in a drop of PBS. They were then immediately used for subsequent staining.

### 4.4. Thioflavin S or Congo Red Staining of Eggs

The eggs were stained using either 1% Thioflavin S (Sigma-Aldrich, Burlington, MA, USA) in 70% ethanol, in a humid chamber, for 5 min at RT (with pre-dehydration in 70% ethanol), or with a 1% aqueous solution of CR (Reanal, Budapest, Hungary) for 10 min at RT. After staining, the preparations were washed three times for 5 min in either 70% ethanol or PBS, respectively, rehydrated (in the case of Thioflavin S) and mounted in an antifade medium containing 1% DABCO (1,4-diazabicyclo[2.2.2]octane) for further microscopy analysis. The preparations were covered with a clean coverslip, and the edges of the coverslip were sealed with nail polish. The slides were stored at 4 °C.

### 4.5. Immunohistochemistry of Eggs

To study the s36 protein, polyclonal antibodies were obtained against the recombinant protein produced and purified from *E. coli* (subsection “Recombinant s36 protein production and purification”). To study the s38 protein, polyclonal antibodies to the peptide CSAVNHPPLVVKPAPV were obtained (Almabion, Voronezh, Russia).

To reduce nonspecific fluorescence, the preparations of eggs were blocked in 1% bovine serum albumin (BSA) in PBS for 1 h at 37 °C. The eggs were then incubated overnight with the primary rabbit anti-s36 antibody or anti-s38 antibody (1:1000; Almabion, Voronezh, Russia) at 4 °C, washed three times in PBS with 0.1% Tween-20, and incubated for 1 h at 37 °C with the secondary Alexa Fluor^®^ 647 Goat Anti-Rabbit antibody (1:1500, Thermo Fisher Scientific, Waltham, MA, USA). After incubation with the secondary antibodies and three washes in PBS with 0.1% Tween-20, the preparations were either used for Thioflavin S staining or mounted in an antifade medium containing 1% DABCO for further cytological analysis. The preparations were covered with a clean coverslip, and the edges of the coverslip were sealed with nail polish. The slides were then stored at 4 °C.

### 4.6. Microscopy

The preparations were analyzed using a TCS SP5 confocal laser scanning microscope (Leica Microsystems GmBH, Wetzlar, Germany) with Leica Application Suite X 3.3.0.16799 software or a Leica DM6000B fluorescent microscope (Leica Microsystems GmBH, Wetzlar, Germany) with Leica QWin standard V. 3.2.0 software. The preparations stained with CR were analyzed using a Leica DMI6000B inverted microscope with Leica Application Suite software (https://www.leica-microsystems.com/products/microscope-software/p/leica-application-suite/, accessed on 9 May 2024). Adobe Photoshop CS5 (Adobe Systems, San Jose, CA, USA) was used for figure assembling.

### 4.7. Immunochemical Analysis of the s36 Protein Aggregation

Fly ovaries were homogenized using a cryogenic laboratory mill as described above (section “Proteomic screening and identification of proteins forming amyloid-like aggregates”). Homogenized ovarian tissue was then suspended in Lysis buffer (50 mM Tris-HCl at pH 7.6, 150 mM NaCl, 10 mM PMSF, 10 mM EDTA) and centrifuged at 805× *g* for 5 min at 4 °C. Normalization to total protein was performed using the Qubit method. In the next step, the samples were separated into soluble and insoluble fractions by centrifugation. Proteins from the soluble and insoluble fractions were applied to the gel and after electrophoresis and being transferred to a PVDF-membrane were detected using antibodies to the s36 protein. Analysis of the ratio of tubulin or actin in different fractions was not included in the objectives of the work. A detailed description of the procedures is provided below. The clarified lysate was then centrifuged at 75,000× *g* for 50 min at 4 °C. The supernatant fraction was transferred to a new tube, and an equal volume of Lysis buffer was added to the precipitate. To denature the proteins, supernatant and pellet fractions were incubated in an SDS-PAGE loading buffer for 15 min at 95 °C. SDS-PAGE was performed in a 10% polyacrylamide gel in a Mini-PROTEAN 3 Cell chamber (Bio-Rad, Hercules, CA, USA). The proteins were transferred to a PVDF Hybond-P Western blotting membrane (Amersham, Buckinghamshire, UK) in Transfer buffer (25 mM Tris-HCl at pH 8.3, 192 mM glycine, 20% EtOH) using a Mini-PROTEAN 3 Cell transfer module (Bio-Rad, Hercules, CA, USA). The molecular weight marker RainBow^TM^ (Amersham, Buckinghamshire, UK) was used for protein size determination. The proteins were detected with the primary rabbit anti-s36 antibody or anti-s38 antibody (1:10,000; Almabion, Voronezh, Russia) and the secondary Goat Anti-Rabbit IgG H&L antibody (ab205718; HRP) (1:80,000; Abcam, Cambridge, UK). Chemiluminescence detection was performed using the Amersham ECL Prime Western Blotting Detection Reagent (GE Healthcare, Chicago, IL, USA), according to the manufacturer’s recommendations, on the ChemiDoc^TM^ XRS+ Imaging System (Bio-Rad, Hercules, CA, USA).

Normalization to total protein was performed using the Qubit method. A comparative analysis of the ratio of the s36 protein in fractions was carried out based on the signal intensity of three Western blots. Densitometric quantification of band intensities was performed using ImageJ software version 1.8.0-112 (https://imagej.net/ij/, accessed on 5 November 2024). A statistical comparison of three biological replicates was performed using Student’s *t*-test, with *p* ≤ 0.05. RStudio software (version 2023.06.1) was used for statistical analysis and data visualization.

### 4.8. Genomic DNA Extraction, Whole-Genome Sequencing, and Analysis

For genomic DNA extraction, 30 anesthetized female flies of the Oregon-R strain and female flies homozygous for the *Cp36^dec2−1^* chromosomal rearrangement were collected in a tube and frozen at −80 °C. Genomic DNA was isolated using a standard protocol [34]. For whole-genome sequencing, DNA libraries were prepared using the NEBNext^®^ Ultra^TM^ II DNA Library Prep Kit for Illumina according to the manufacturer’s recommendations (New England Biolabs, Ipswich, MA, USA) from 100 ng of prepared DNA. The quality of libraries was tested by capillary electrophoresis using a QIAxcel Advanced system (QIAGEN, Hilden, Germany), and the median peak length of the analyzed libraries was 375 bp. Library preparation and pair-end sequencing of 150 nucleotides of inserts were performed in the “Bio-Bank” Resource Center of St. Petersburg State University Research Park using a HiSeq2500 (Illumina, San Diego, CA, USA). The raw sequencing reads in fastq format were preprocessed with fastp software (version 0.23.2) [35] to remove adapters and low-quality bases. The processed reads were aligned to *D. melanogaster* reference genome version 6.49 with the bwa mem algorithm [36] using default parameters. As a result, 98.36% of 23,527,401 reads and 92.85% of 34,745,720 reads were aligned and properly paired for the wild-type and mutant samples, respectively. Two alternative structural change analysis tools were used, namely Griss (version 2.13.2) [37] and Delly (version 1.1.6) [38]. The whole-genome sequencing data were deposited into the Sequence Read Archive (SRA) database, corresponding BioProject ID: PRJNA1031583.

### 4.9. Clarification of the CG33223 Gene Deletion

The coordinates for genomic location and the size of the detected deletion in the *CG33223* gene were clarified using Sanger sequencing. The *CG33223* gene fragment amplification was carried out by PCR with Taq-polymerase (ThermoFisher Scientific, Waltham, MA, USA) using the genomic DNA as a template (genomic DNA extraction was described above) and the following PCR primers: *For_CG33223* (forward), 5′-GGATGCGGGGAATAAACATAC-3′; *Rev_CG33223* (reverse), 5′-CATCGTCACCTGCGTTGAAG-3′. PCR products (355 bp and 992 bp for wild type and mutant, respectively) were analyzed by agarose gel electrophoresis. The PCR product of the *CG33223* gene with the detected deletion was sequenced using the Sanger method with the above primers. The Sanger sequencing data were deposited into the GenBank database, corresponding accession number: PP658205.

### 4.10. Cloning of the Cp36 Gene and Sanger Sequencing

Ovary homogenates of *D. melanogaster* were obtained using a cryogenic laboratory mill as described above. Total RNA was extracted from ovarian homogenates using the TRIzol^TM^ reagent (Thermo Fisher Scientific, Waltham, MA, USA) according to the manufacturer’s protocol. cDNA synthesis using oligo(dT)12-18 was performed with SuperScript^TM^ III Reverse Transcriptase (Thermo Fisher Scientific, Waltham, MA, USA) according to the manufacturer’s protocol. cDNA was further used for *Cp36* amplification by PCR with Taq-polymerase (ThermoFisher Scientific, Waltham, MA, USA) using the following PCR primers: *For_ch36_cDNA* (forward), 5′-ATGCAACTCGGTCTCTGGTT-3′ and *Rev_ch36_cDNA* (reverse), 5′-TTAGTAGTTGGGCTGGCCAT-3′. The *Cp36* gene was cloned into pJET 1.2 using a CloneJET PCR Cloning Kit (Thermo Fisher Scientific, Waltham, MA, USA) according to the manufacturer’s protocol and next was sequenced using the Sanger method with the T7 primers. The cDNA sequences of the *Cp36* transcripts from the Oregon-R flies and the flies carrying the *Cp36^dec2−1^* chromosomal rearrangement were compared.

### 4.11. Analysis of Amyloid Properties In Vitro

#### 4.11.1. Recombinant s36 Protein Production and Purification

The fragment of the *Cp36* gene coding for 21–284 aa of s36 (full protein without signal sequence) for expression in *E. coli* was amplified from cDNA with For-s36-CHis (forward; 5′-CGCTCTAGACTGGTGAGCGCTAACTATGGTC-3′) and Rev-s36-CHis (reverse; 5′-CAGAGCTCGTAGTTGGGCTGGCCATAGGG-3′) primers. The obtained amplicon was inserted into the pET303 vector using the T4 DNA Ligase (Thermo Fisher Scientific, Waltham, MA, USA) to obtain the pET303-s36 plasmid. The 6His-tagged s36 protein synthesis was induced with 0.2 mM isopropyl β-D-1-thiogalactopyranoside (IPTG) by incubating NiCo21(DE3) pLysS cells in LB medium overnight at 22 °C. The cells were then disrupted by sonication in 20 mM PBS (pH 8.0) supplemented with 0.7 mM NaCl, 1.5 mM DTT, 1 mM EDTA, and 1.2 mM PMSF. The obtained lysate was centrifuged at 12,879× *g* for 10 min at 4 °C to precipitate cellular debris. Protein from the clarified lysate was further purified by affinity chromatography on Ni-NTA Agarose (Thermo Fisher Scientific, Waltham, MA, USA) according to the manufacturer’s protocol. The purity of the recombinant protein was verified by SDS-PAGE, and the protein concentration was measured using a Qubit^®^ 2.0 Fluorometer (Thermo Fisher Scientific, Waltham, MA, USA). This protein was used for the in vitro fibril formation assay and for obtaining the anti-s36 polyclonal antibodies.

#### 4.11.2. In Vitro Fibril Formation Assay

The purified 6His-tagged s36 protein was transferred onto a 10 kDa Amicon Ultra filter (Sigma-Aldrich, Burlington, MA, USA) to exchange the chromatography buffer to PBS supplemented with 2 mM DTT and a Halt^TM^ Protease Inhibitor Cocktail (Thermo Fisher Scientific, Waltham, MA, USA). The obtained protein solution was incubated at RT under slow rotation up to 5 days to obtain fibrils. Fibril formation was verified by TEM and CR staining.

#### 4.11.3. Sample Preparation for TEM and Fibril Structure Analysis

Negatively stained samples were prepared on a formvar-coated copper grid (Formvar/Carbon Film 10 nm/1 nm thick on Square 300 mesh Copper Grid; Electron Microscopy Sciences, Hatfield, PA, USA). A 10 μL aliquot of the fibril solution was adsorbed to the formvar grid for 1 min, blotted, washed twice with 10 μL of water for 10 s, and then stained with 10 μL of 1% uranyl acetate (Electron Microscopy Sciences, Hatfield, PA, USA) for 1 min. After removal of uranyl acetate, the probes were dried in air. The fibril structure was analyzed using a JEM-2100 HC electron microscope (JEOL, Tokyo, Japan). Adobe Photoshop CS5 (Adobe Systems, San Jose, CA, USA) was used for figure assembling.

#### 4.11.4. Congo Red Staining of Fibrils

A 10 μL aliquot of the fibril solution was put onto a glass microscope slide, air dried, stained with 50 μL of a 1% aqueous solution of CR (Reanal, Budapest, Hungary) for 5 min at RT, washed with water, and covered with a clean coverslip. Slides were analyzed in brightfield and between cross polarizers on the polarized light microscope Biolar PI-PZO (PZO Microscopy, Warsaw, Poland).

### 4.12. Analysis of Amyloid Properties Ex Vivo

An analysis of the amyloid properties of the s36 protein ex vivo was performed using the approach of immunoprecipitation of amyloid fibrils [19,24]. For immunoprecipitation of the s36 protein from *D. melanogaster* ovaries, the anti-s36 antibodies (Almabion, Voronezh, Russia) were bound with the protein A-coated magnetic beads SileksMagX-Protein A (Sileks, Moscow, Russia) in binding buffer (1 × PBS, 0.02% Tween-20, 1 × Halt^TM^ Protease Inhibitor Cocktail [Thermo Fisher Scientific, Waltham, MA, USA]) for 1 h at RT with slow overhead rotation. The prepared magnetic beads with antibodies were then incubated with fly ovarian lysate for 2 h at RT. Protein elution was performed with 125 mM glycine buffer (pH 2.1) for 10 min at RT according to the manufacturer’s protocol (Sileks, Moscow, Russia). After neutralization by 1.5 M Tris-buffer (pH 8.8), the fibrils were sedimented by centrifugation at 436,000× *g* for 1.5 h at 4 °C, stained with CR dye, and analyzed by brightfield, polarization, and TEM microscopy as described above (section “Analysis of amyloid properties in vitro”).

### 4.13. Bioinformatic Analysis of s36 Gene and Protein Sequences

The protein sequence AAF46382.1 corresponding to the gene *Cp36* (NC_004354.4) was obtained from the NCBI data repository (https://www.ncbi.nlm.nih.gov/search/, accessed on 2 July 2024). The performed scan for potential amyloidogenic regions in the s36 protein sequence was carried out using the ArchCandy-1.0 algorithm [30]. The threshold for the search was set to 0.578. The protein orthologs were identified using the OrthoDB database (https://www.orthodb.org/, accessed on 2 July 2024) and the BLAST algorithm (https://blast.ncbi.nlm.nih.gov/Blast.cgi, accessed on 2 July 2024) with a threshold for significant hits (E-value) of 0.05.

## Figures and Tables

**Table 1:** Chorion proteins identified with PSIA-LC MALDI in the Oregon-R strain of *D. melanogaster* ovaries.

Protein	Score 1	Score 2 *
Chorion protein s36	1531.6	903.1
Chorion protein s19	1085.8	654.9
Chorion protein s38	836.6	103.5
Chorion protein s15	720.1	439.5
Chorion protein s18	667.8	−
Chorion protein s16	532.1	−

Score of mass spectrometry is determined according to WARP-LC software (version 1.3). * Corresponds to the results of proteomic screening with chitinase treatment.

## Data Availability

The whole-genome sequencing data were deposited into the SRA under accession number PRJNA1031583. The Sanger sequencing data were deposited into the GenBank database under accession number PP658205.

## Supplementary Materials

The following supporting information can be downloaded at https://www.mdpi.com/article/10.3390/ijms252312499/s1.
