# Supplementary figures and images for: Amyloid Fibrils of the s36 Protein Modulate the Morphogenesis of Drosophila melanogaster Eggshell
Source: Int J Mol Sci. 2024 Nov 21;25(23):12499. doi: 10.3390/ijms252312499 (PMC11641622; doi:10.3390/ijms252312499)

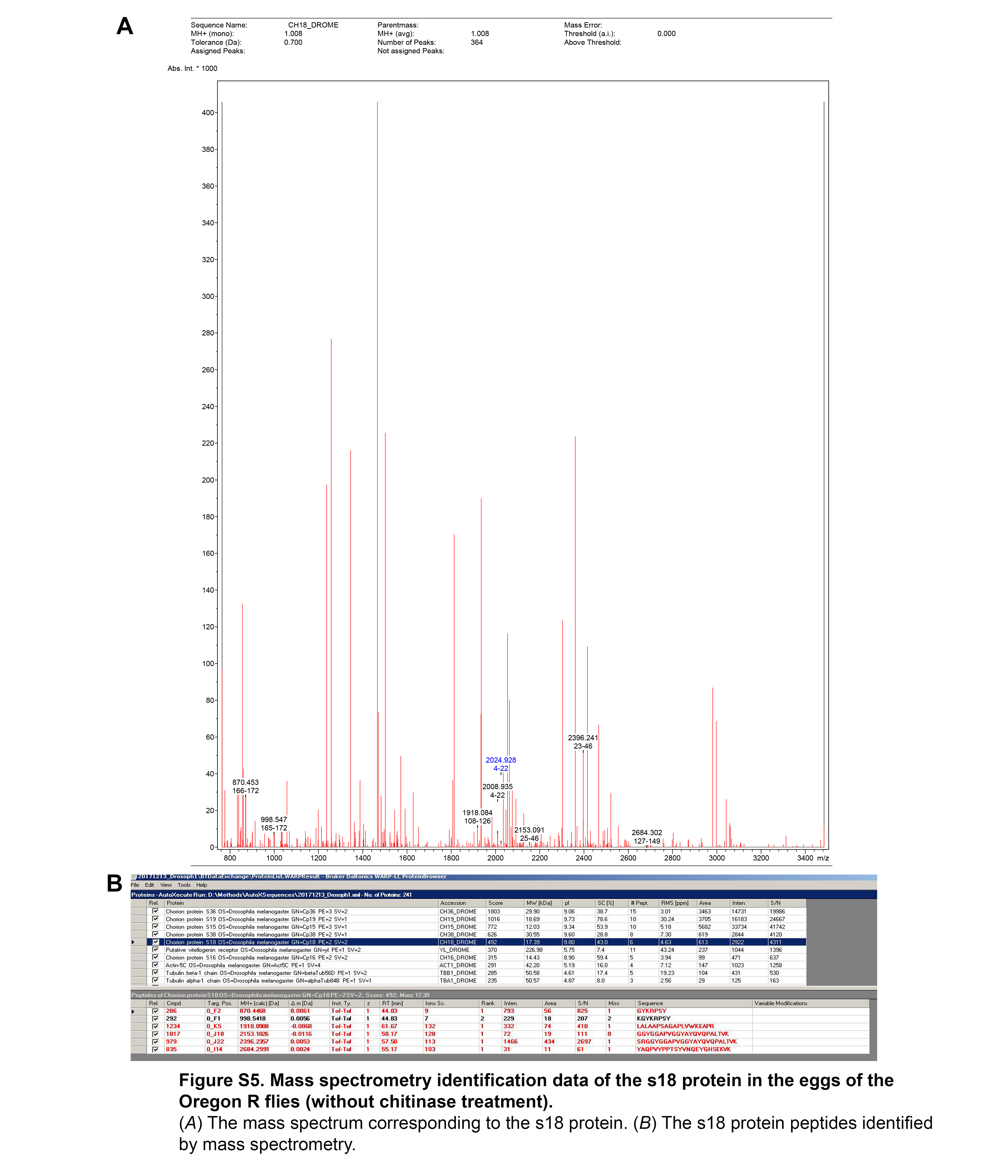

Supplement: Supplementary file 1 [file ijms-25-12499-s001.zip › Figure S5.tif]

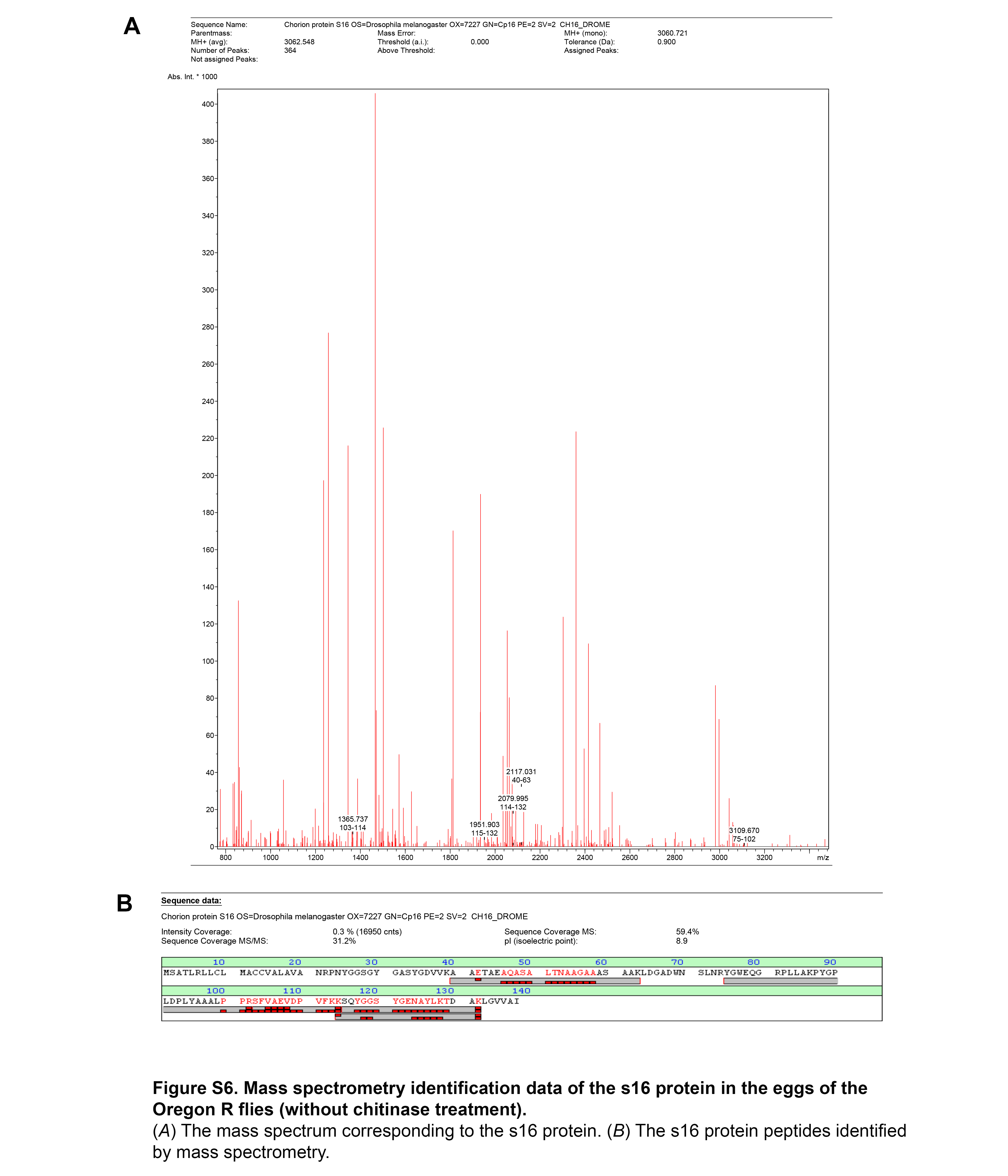

Supplement: Supplementary file 1 [file ijms-25-12499-s001.zip › Figure S6.tif]

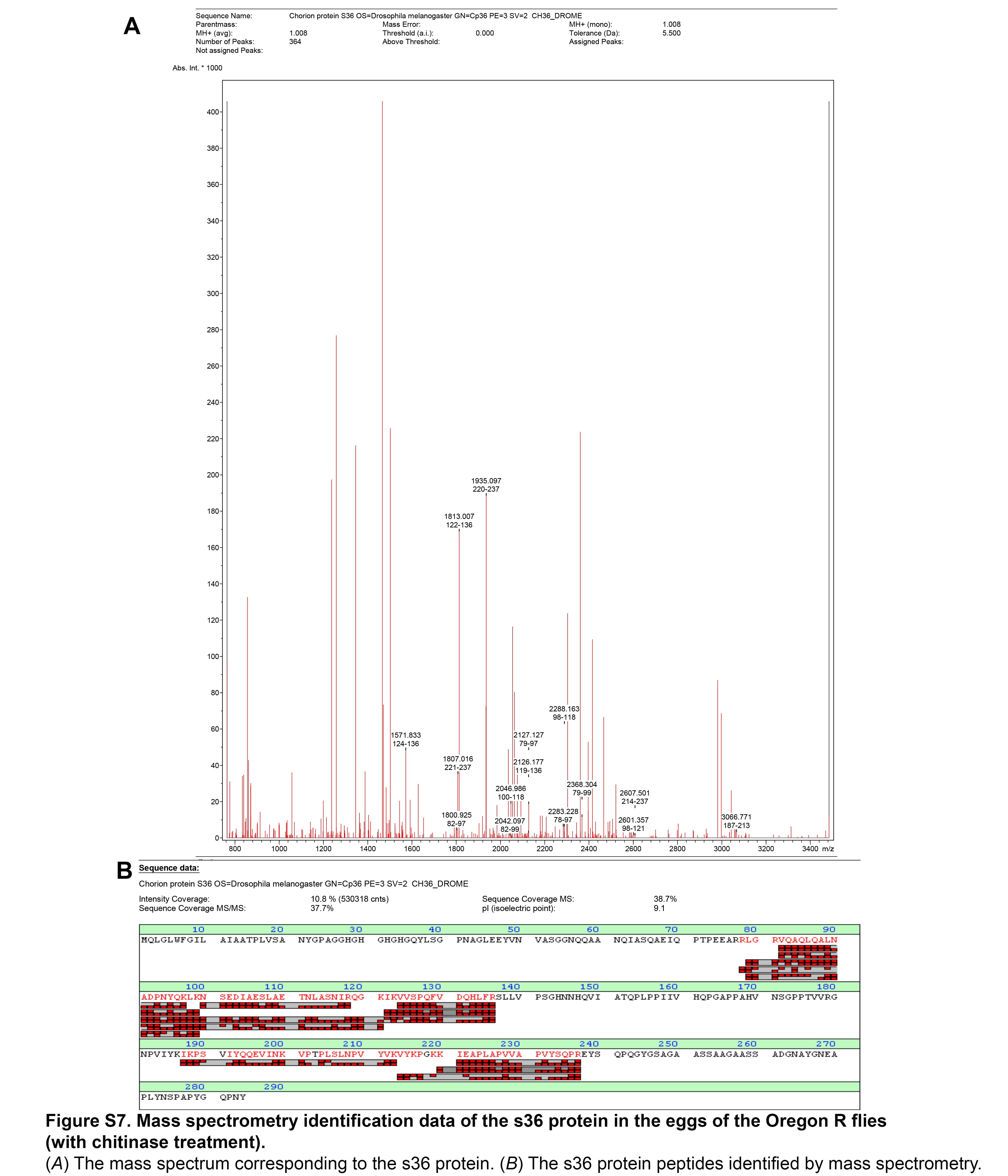

Supplement: Supplementary file 1 [file ijms-25-12499-s001.zip › Figure S7.tif]

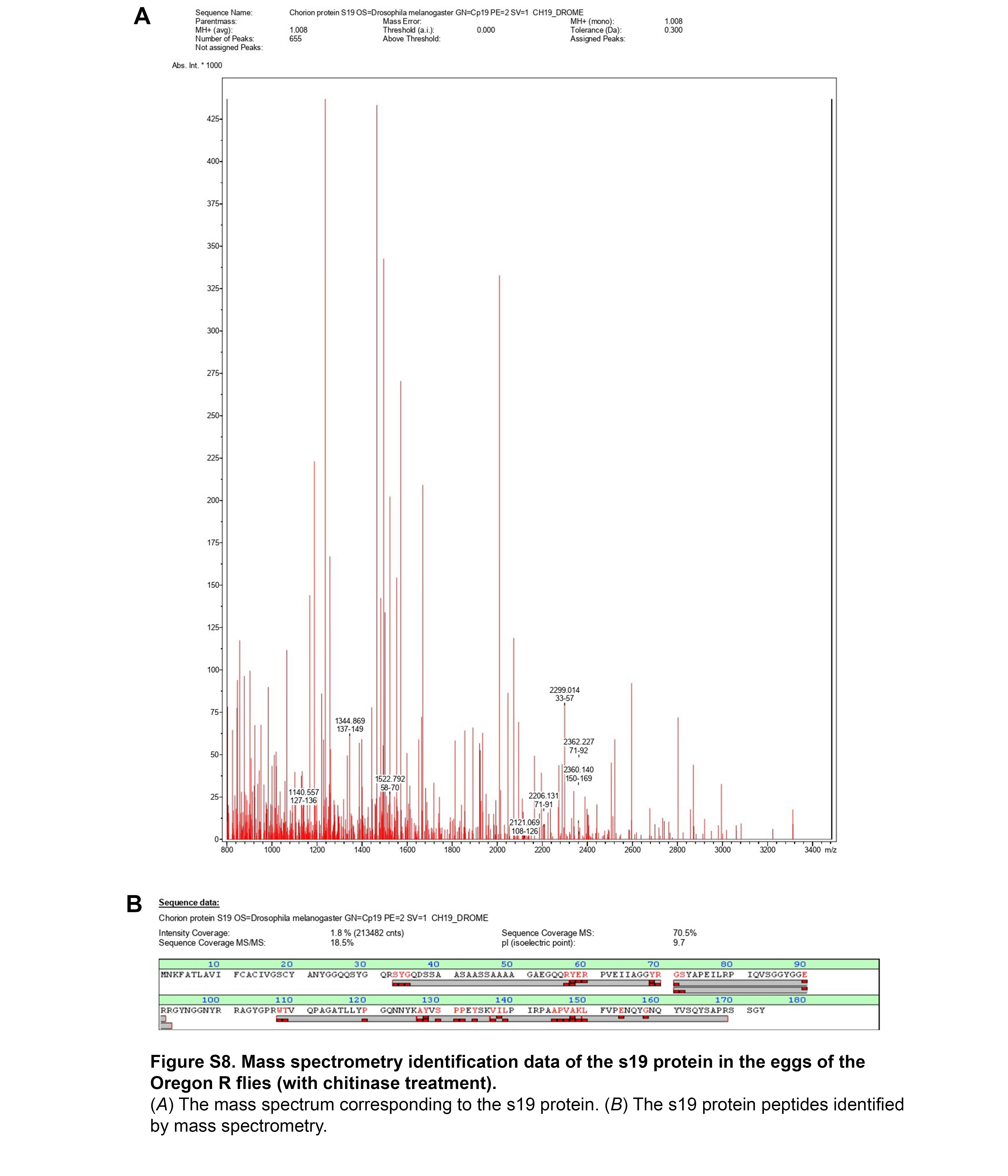

Supplement: Supplementary file 1 [file ijms-25-12499-s001.zip › Figure S8.tif]

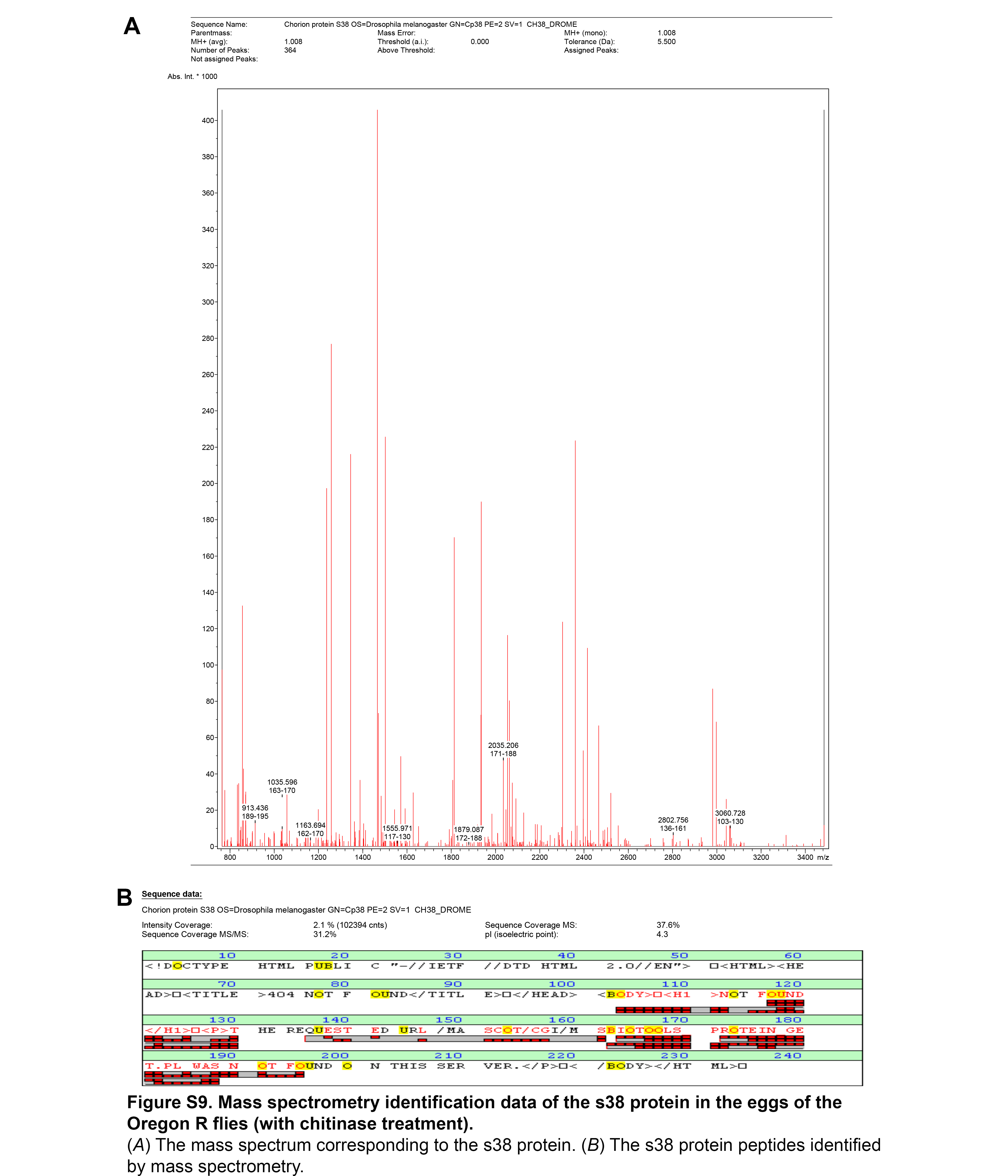

Supplement: Supplementary file 1 [file ijms-25-12499-s001.zip › Figure S9.tif]

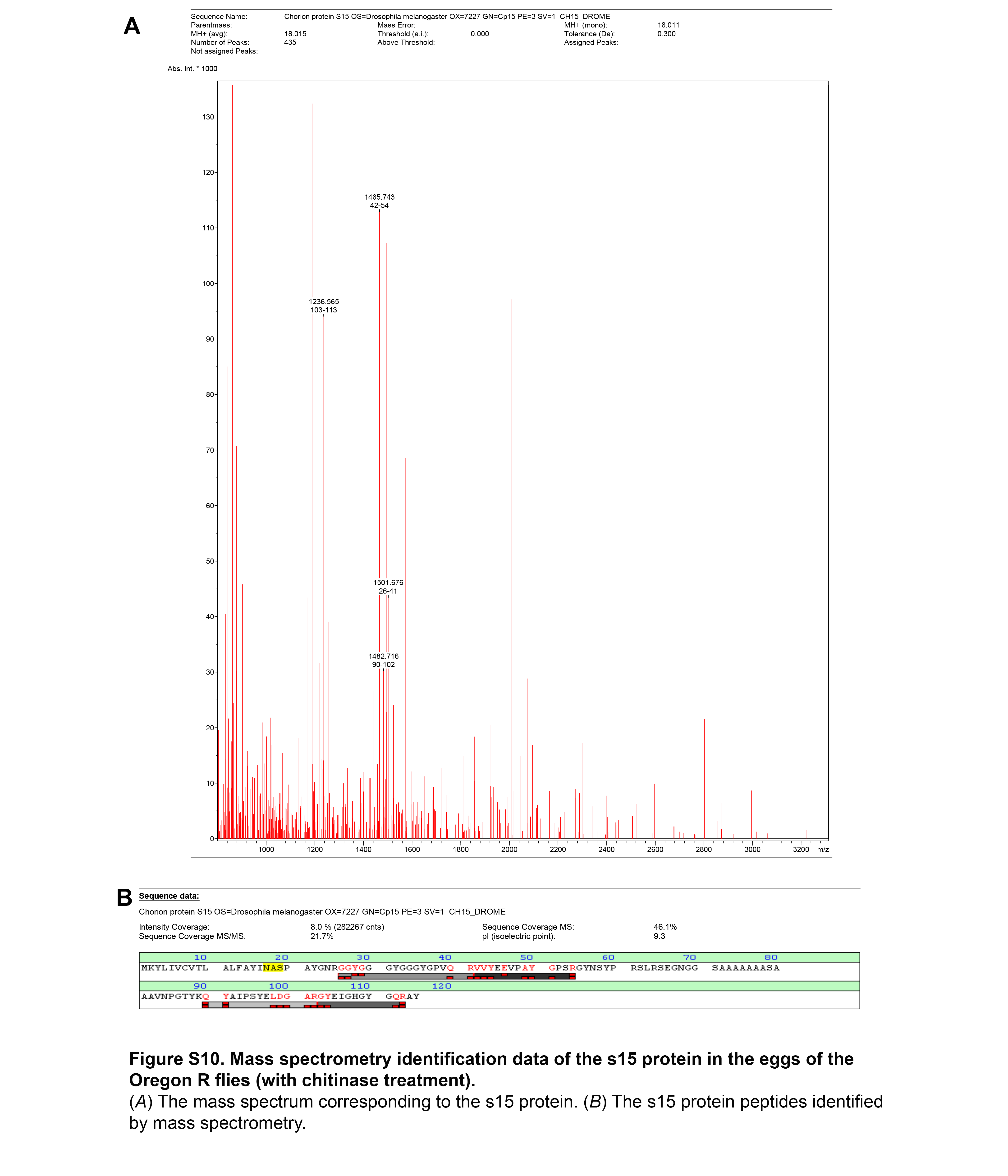

Supplement: Supplementary file 1 [file ijms-25-12499-s001.zip › Figure S10.tif]

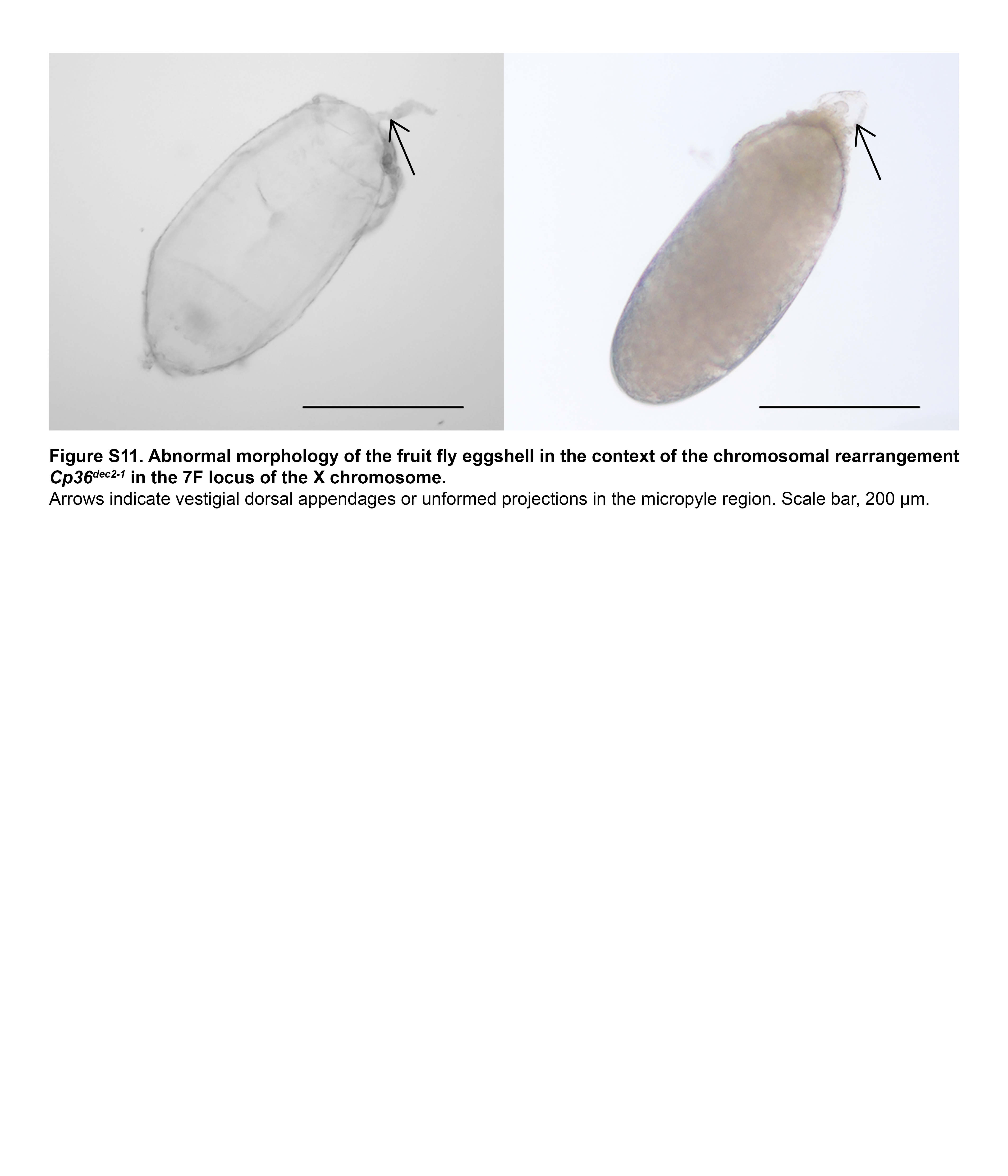

Supplement: Supplementary file 1 [file ijms-25-12499-s001.zip › Figure S11.tif]

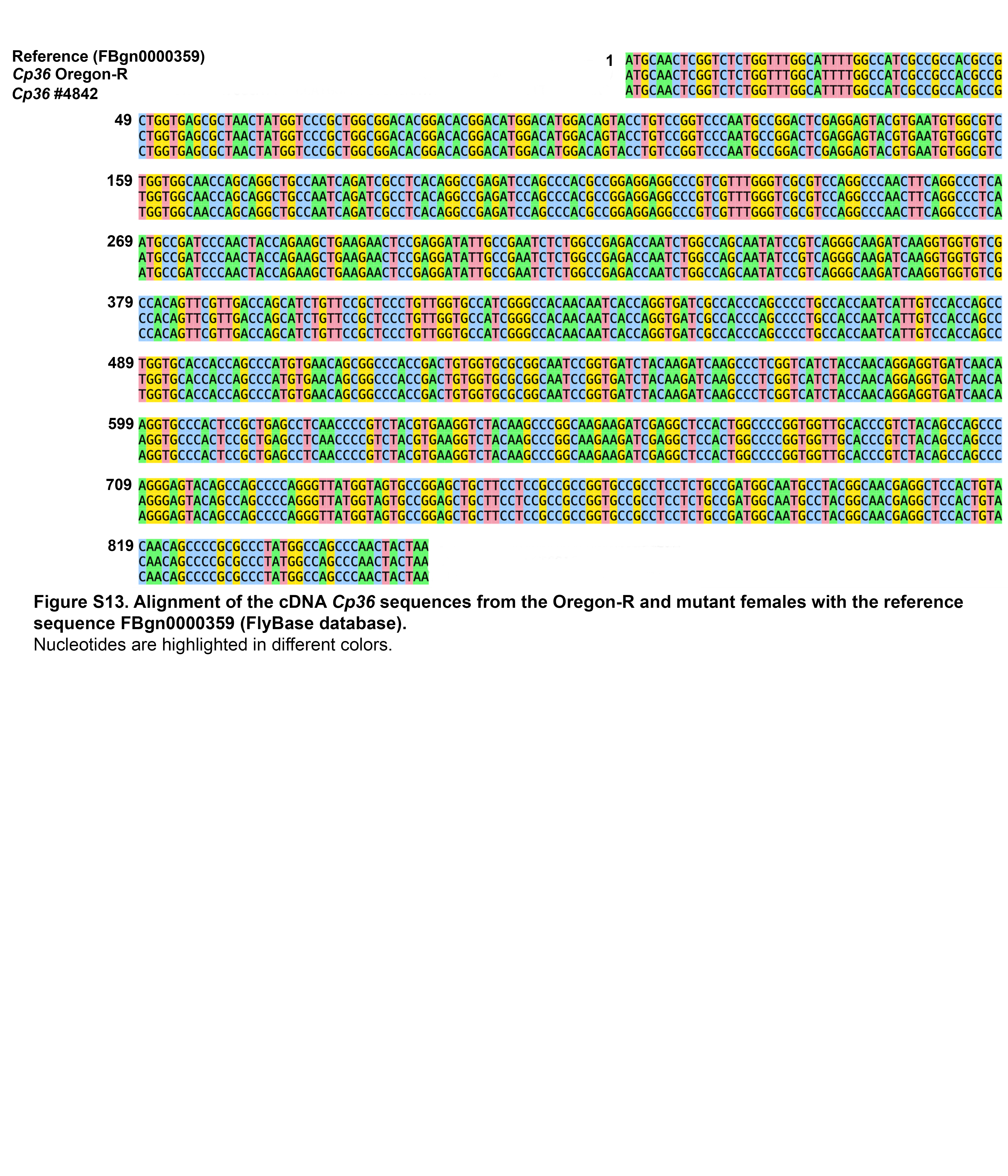

Supplement: Supplementary file 1 [file ijms-25-12499-s001.zip › Figure S13.tif]

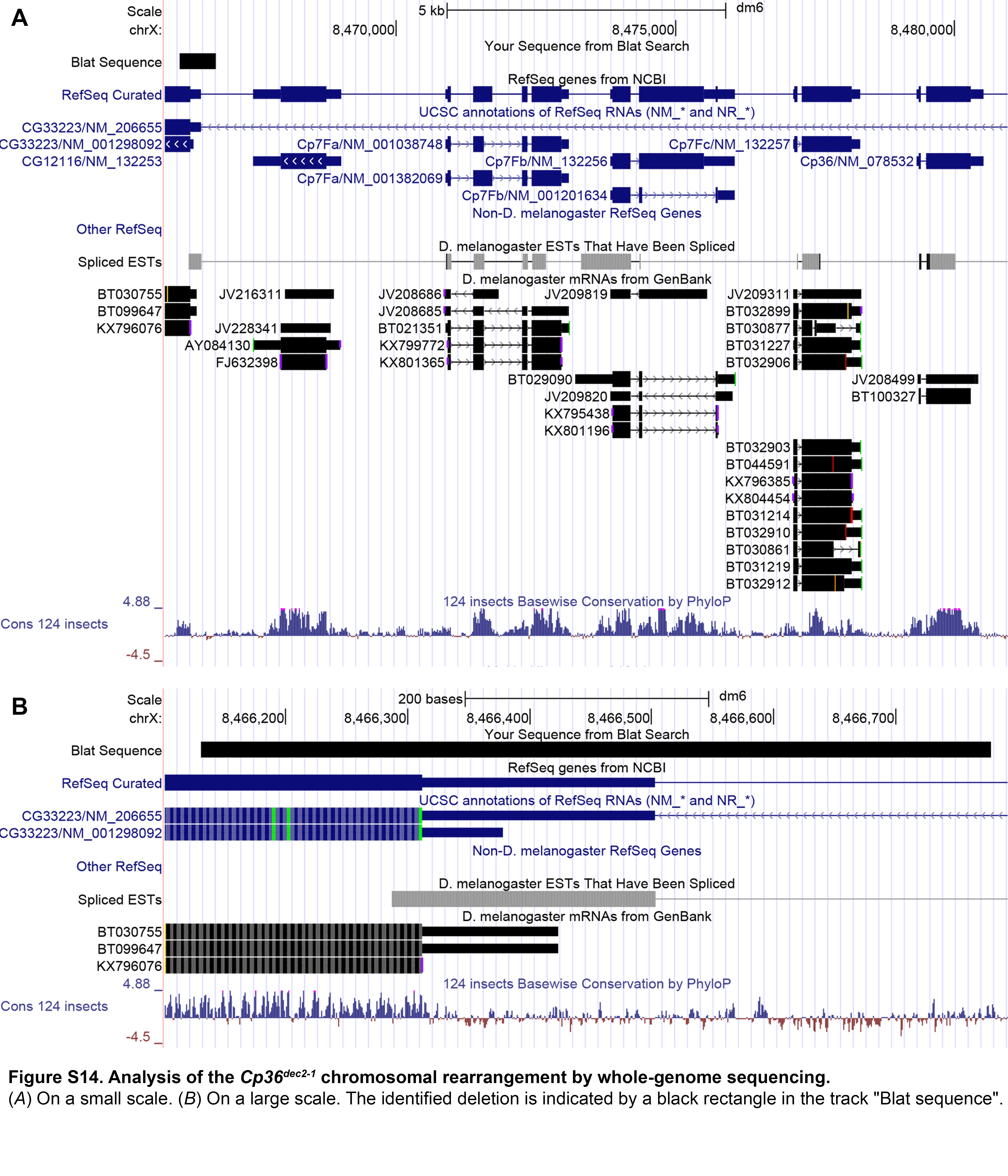

Supplement: Supplementary file 1 [file ijms-25-12499-s001.zip › Figure S14.tif]

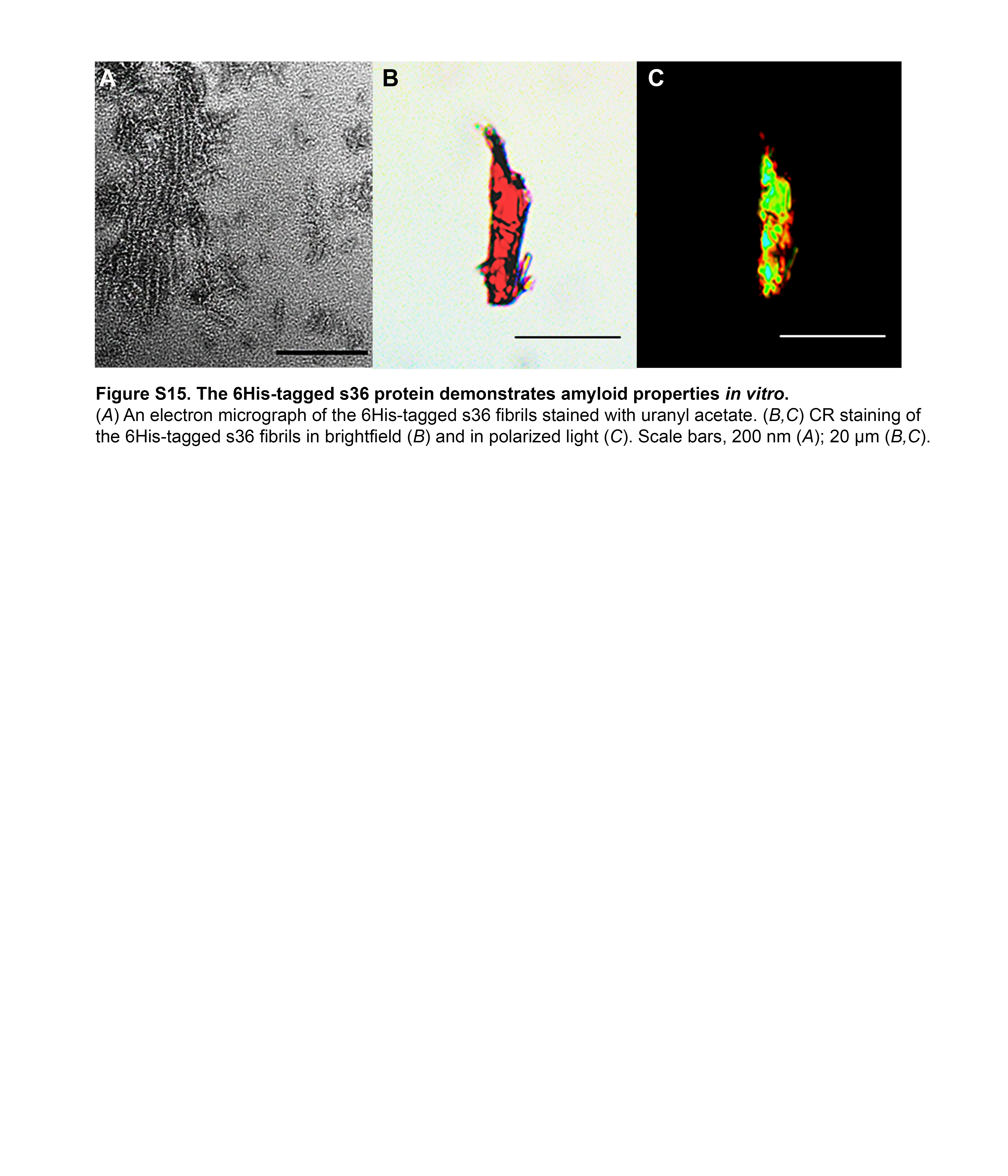

Supplement: Supplementary file 1 [file ijms-25-12499-s001.zip › Figure S15.tif]

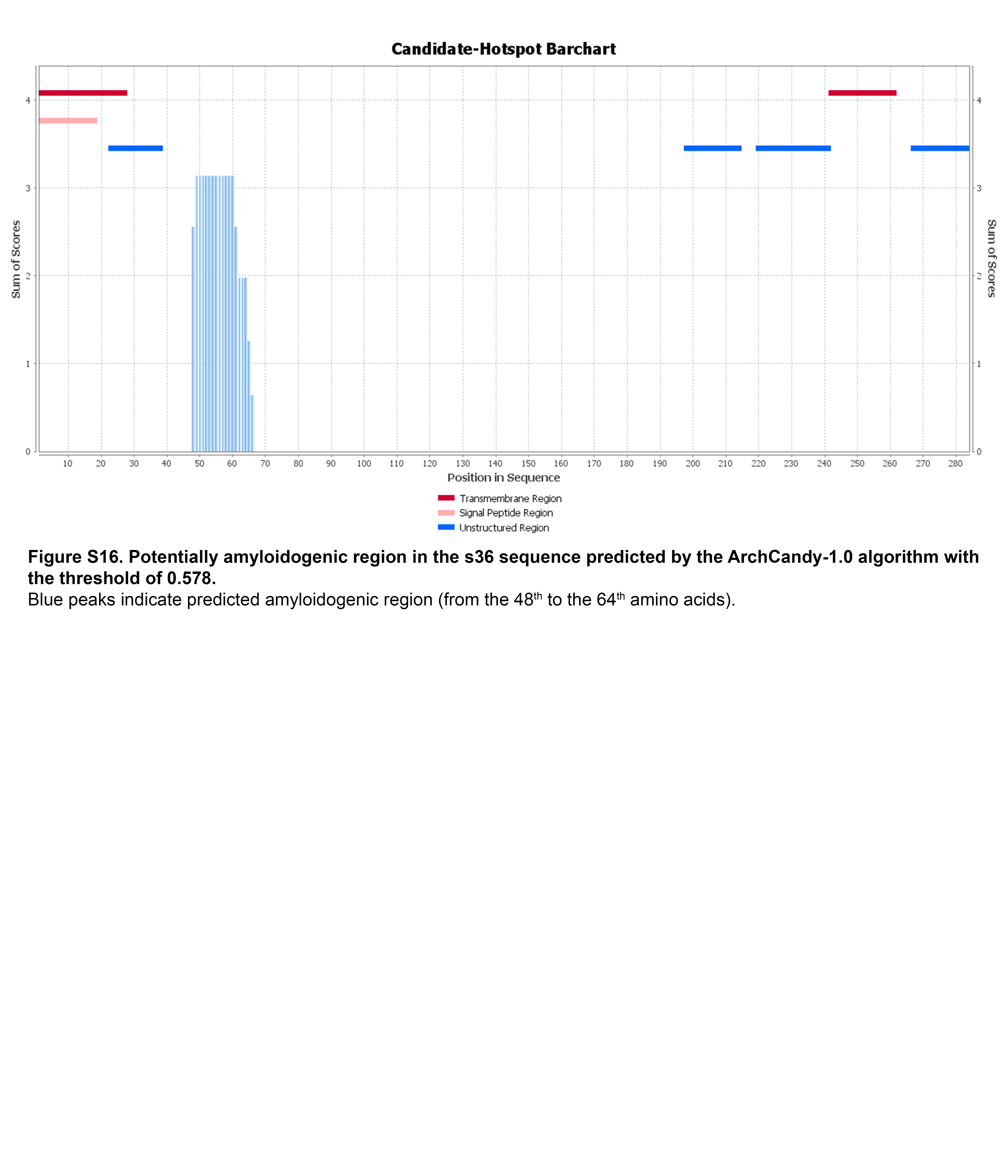

Supplement: Supplementary file 1 [file ijms-25-12499-s001.zip › Figure S16.tif]

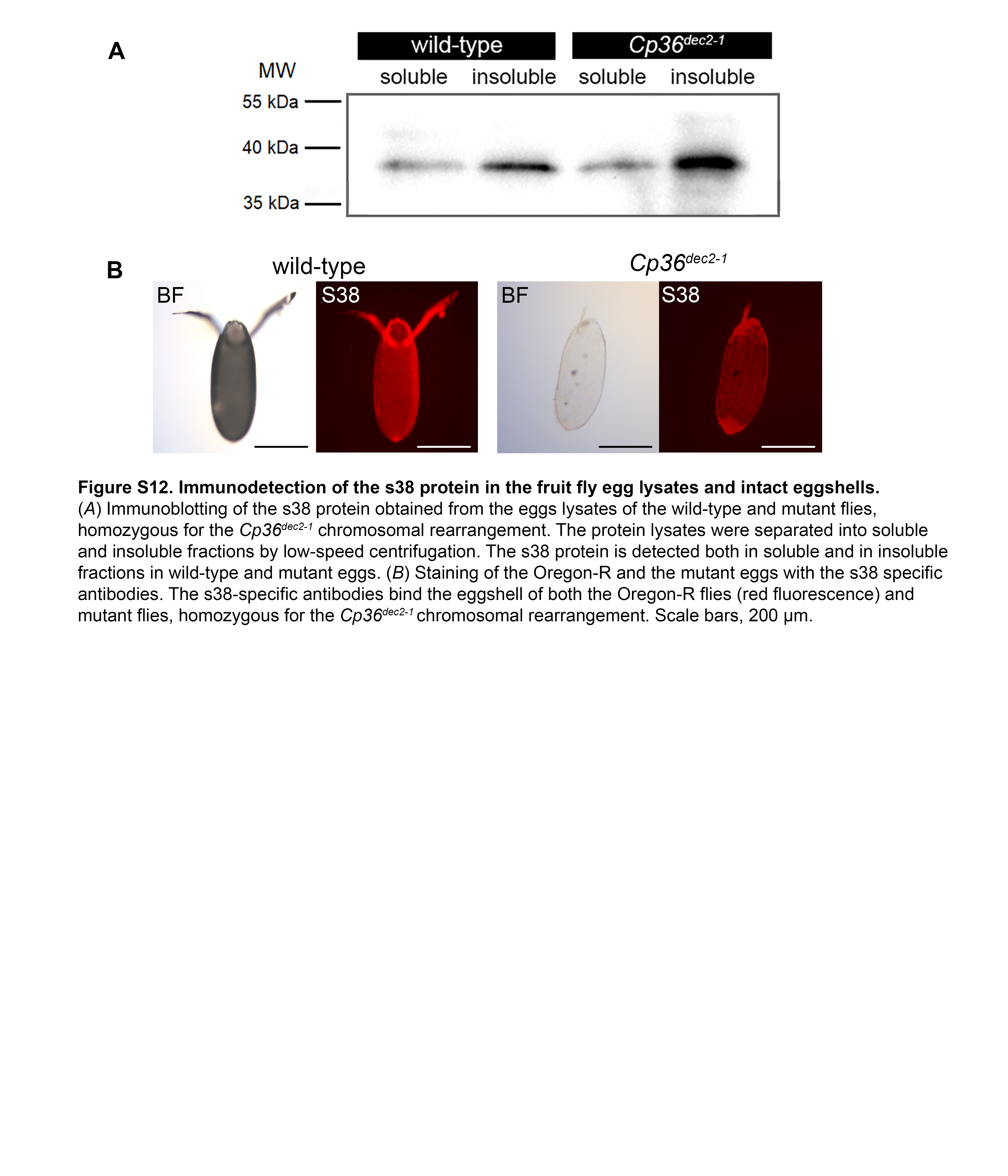

Supplement: Supplementary file 1 [file ijms-25-12499-s001.zip › Figure S12.tif]

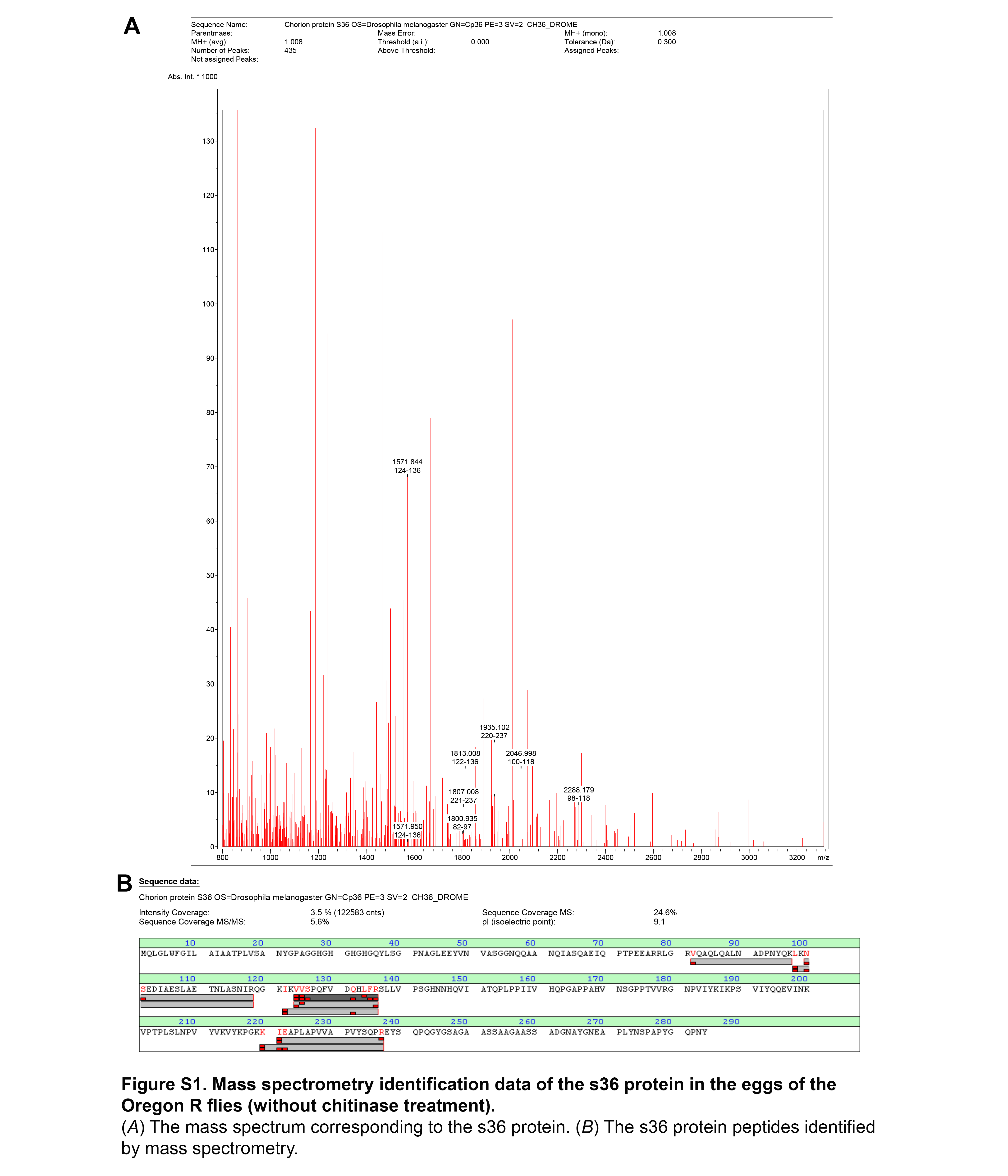

Supplement: Supplementary file 1 [file ijms-25-12499-s001.zip › Figure S1.tif]

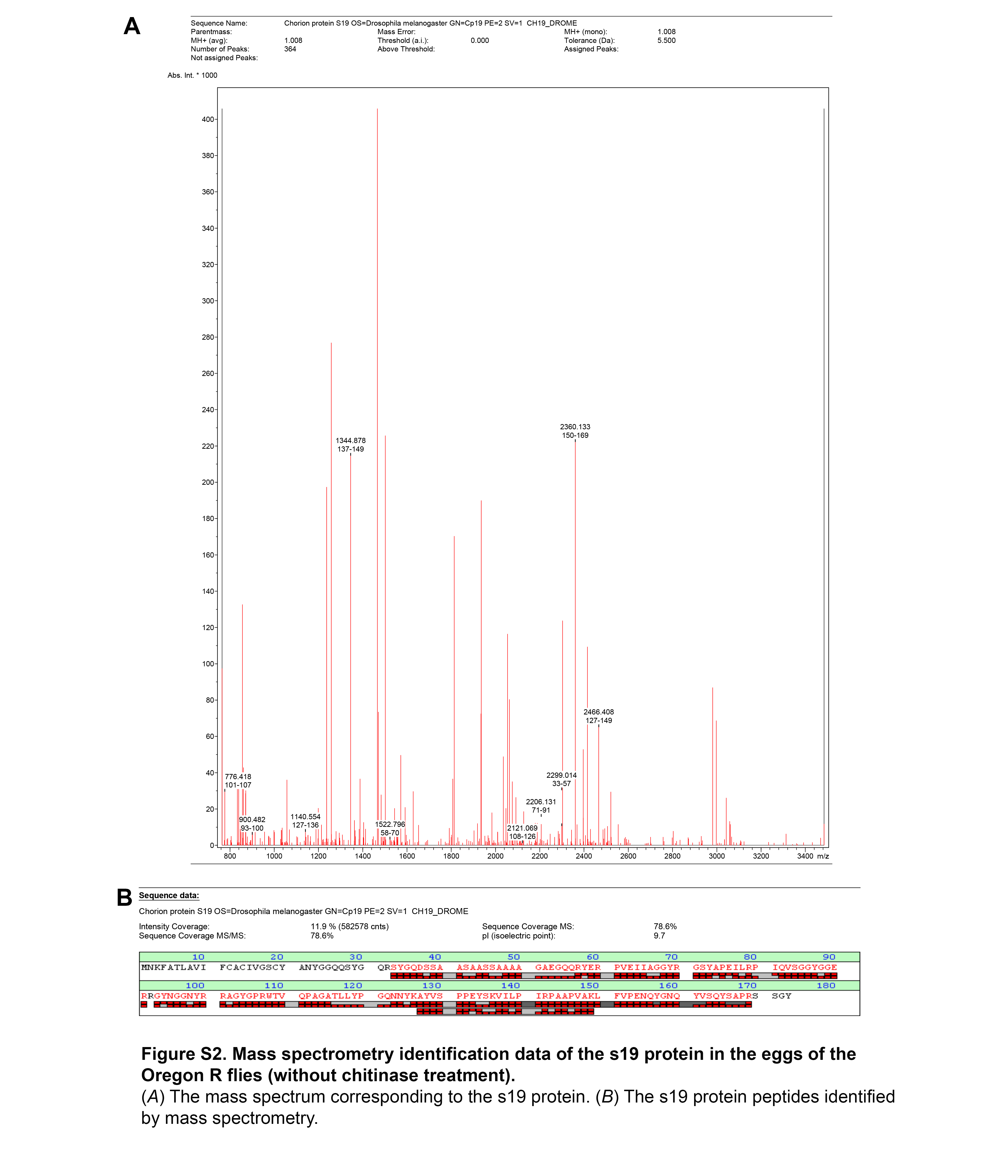

Supplement: Supplementary file 1 [file ijms-25-12499-s001.zip › Figure S2.tif]

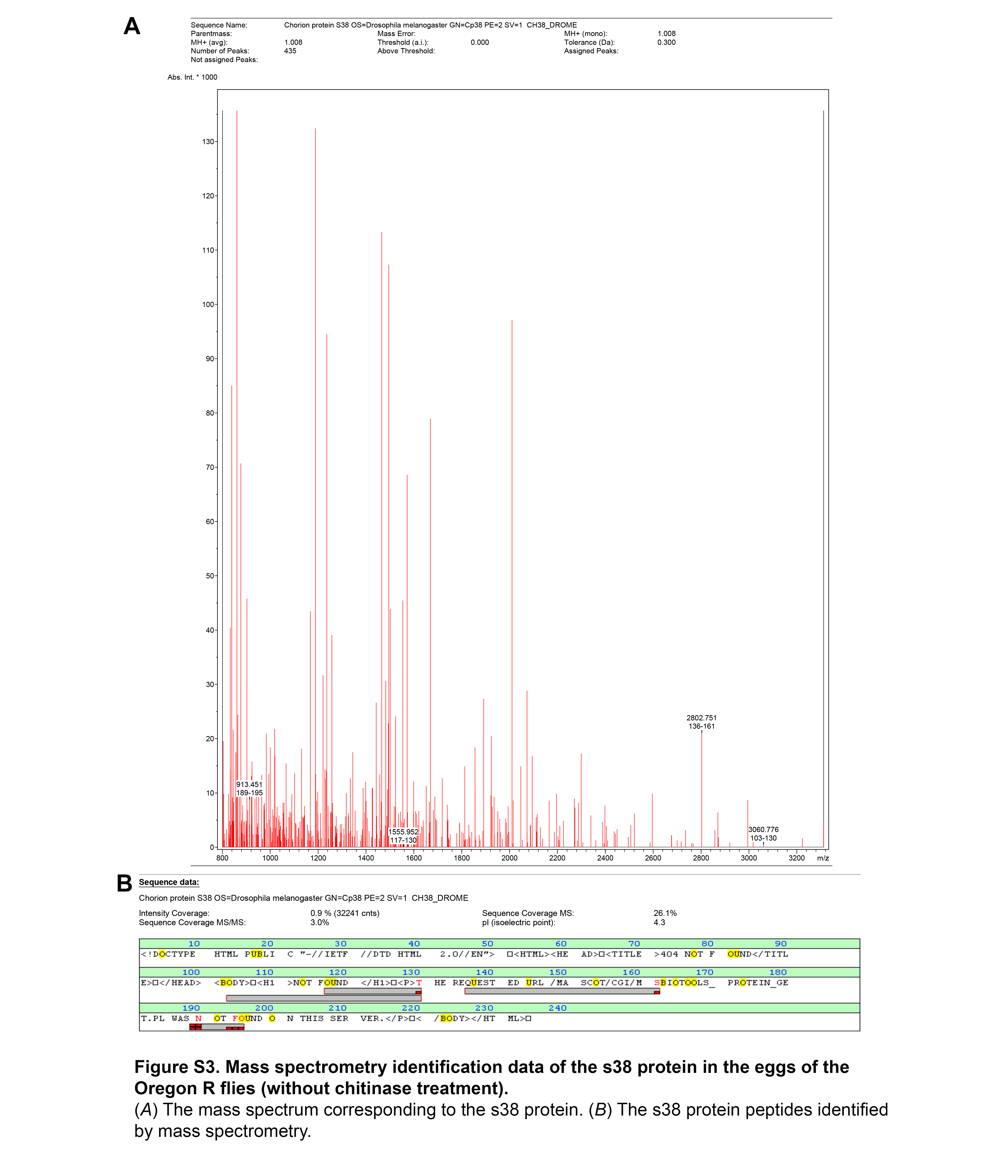

Supplement: Supplementary file 1 [file ijms-25-12499-s001.zip › Figure S3.tif]

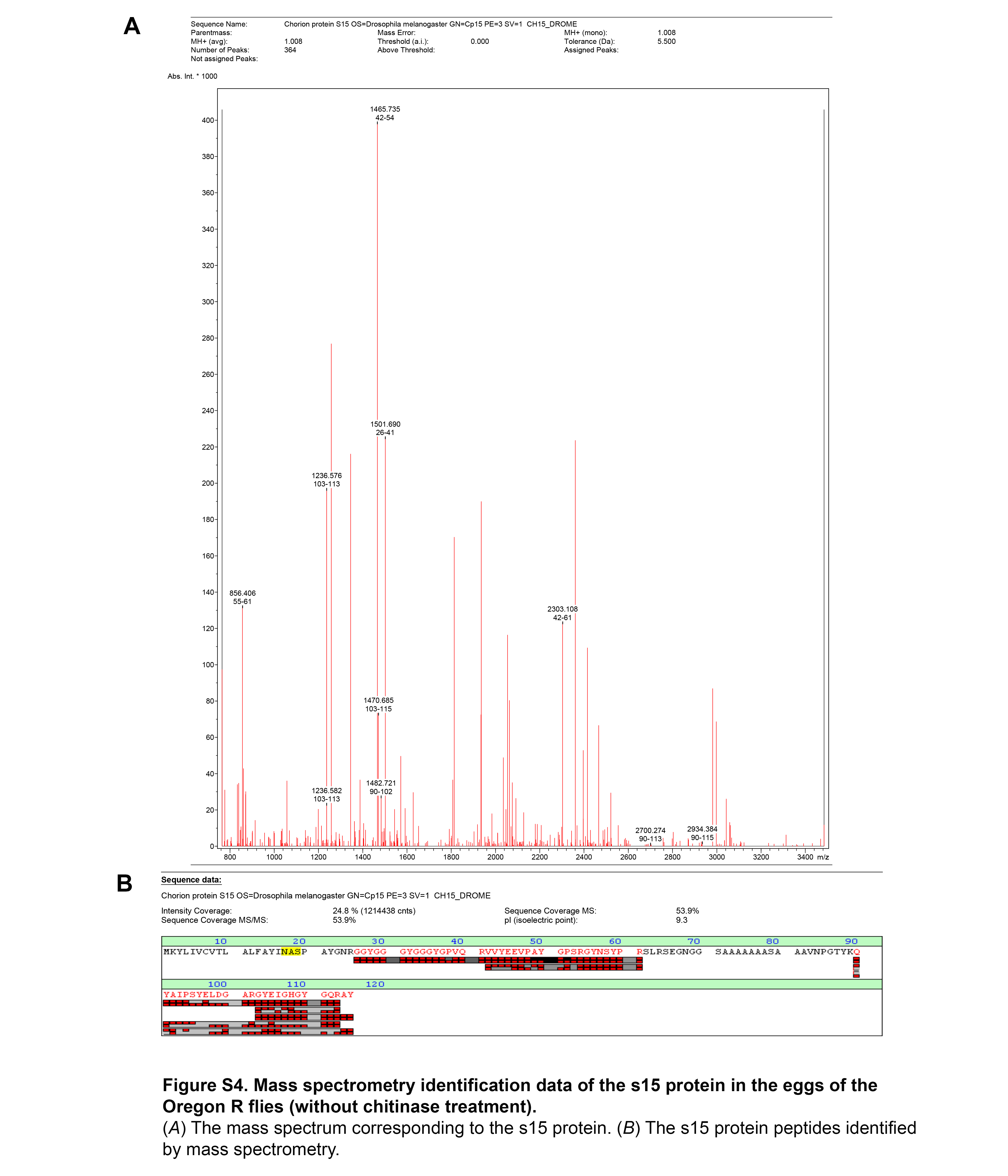

Supplement: Supplementary file 1 [file ijms-25-12499-s001.zip › Figure S4.tif]
